# Avalanches during epithelial tissue growth; Uniform Growth and a drosophila eye disc model

**DOI:** 10.1371/journal.pcbi.1009952

**Published:** 2022-03-18

**Authors:** George Courcoubetis, Chi Xu, Sergey V. Nuzhdin, Stephan Haas

**Affiliations:** 1 Department of Physics and Astronomy, University of Southern California, Los Angeles, California, United States of America; 2 Department of Biology, University of Southern California, Los Angeles, California, United States of America; Oxford, UNITED KINGDOM

## Abstract

Epithelial tissues constitute an exotic type of active matter with non-linear properties reminiscent of amorphous materials. In the context of a proliferating epithelium, modeled by the quasistatic vertex model, we identify novel discrete tissue scale rearrangements, i.e. cellular rearrangement avalanches, which are a form of collective cell movement. During the avalanches, the vast majority of cells retain their neighbors, and the resulting cellular trajectories are radial in the periphery, a vortex in the core. After the onset of these avalanches, the epithelial area grows discontinuously. The avalanches are found to be stochastic, and their strength is correlated with the density of cells in the tissue. Overall, avalanches redistribute accumulated local spatial pressure along the tissue. Furthermore, the distribution of avalanche magnitudes is found to obey a power law, with an exponent consistent with sheer induced avalanches in amorphous materials. To understand the role of avalanches in organ development, we simulate epithelial growth of the *Drosophila* eye disc during the third instar using a computational model, which includes both chemical and mechanistic signaling. During the third instar, the morphogenetic furrow (MF), a ~10 cell wide wave of apical area constriction propagates through the epithelium. These simulations are used to understand the details of the growth process, the effect of the MF on the growth dynamics on the tissue scale, and to make predictions for experimental observations. The avalanches are found to depend on the strength of the apical constriction of cells in the MF, with a stronger apical constriction leading to less frequent and more pronounced avalanches. The results herein highlight the dependence of simulated tissue growth dynamics on relaxation timescales, and serve as a guide for *in vitro* experiments.

## Author summary

Epithelial tissues have interesting properties. They are often described as viscoelastic materials, behaving like an elastic solid on short time scales and as a liquid on long time scales. When tissues proliferate, cell division requires the cells to rearrange and displace each other. To relieve proliferative stress, cells are equipped with machinery that allows them to respond to mechanical cues and rearrange. Herein, we perform computational simulations, based on the vertex model, to investigate epithelial growth. In our simulations, when the tissue size reaches thousands of cells, we observe the prevalence of stochastic step-like growth discontinuities. These newly identified events, which we term avalanches, involve the collective rearrangement of all cells in the tissue, and they are accompanied with localized cellular pressure redistribution and a collective net radial displacement of cells. We study their properties in the simple setting of a spatially uniform and time independent growth model and in the context of an experimentally calibrated Drosophila eye disc growth model. Utilizing the eye disc growth model, we also investigate the relationship between localized cellular mechanical deformations and global growth dynamics. We conclude that avalanches constitute a macroscopic pathway for epithelial tissues to release accumulated proliferative stress when local processes are insufficient.

## Introduction

Ensembles of epithelial cells give rise to solid and liquid structural properties. Epithelial tissues are often considered viscoelastic materials, which exhibit elastic material properties on short time scales and liquid properties on long time scales [1]. A prevailing notion is that the viscoelastic structure of tissues is a consequence of being in a liquid phase, but in proximity to a transition to a glass phase [2,3]. Notably, growing and space adaptive tissues do not exhibit glassy behavior, since this would imply solid material properties on long time scales [2]. Computational models that use Self Propelled Particle (SPP) models to describe epithelial dynamics capture a density dependent glass transition [2,4–6]. However, such a transition can also be achieved by tuning cell structural parameters and fluctuations [7].

In this computational study, we find that the viscoelastic nature of tissue growth can be accompanied by sequences of cellular rearrangement avalanches. The tissue remains in an elastic solid structural state during growth. As the cells proliferate, the cell density increases until localized pressure pockets redistribute across the tissue, causing large-scale rearrangement events, which we define as avalanches. During this rearrangement, the cells predominantly retain their neighbors and collectively flow radially, leading to a sharp increase in tissue size. The properties of the identified avalanches are investigated in the context of a uniformly growing epithelial tissue and a model of *Drosophila* eye disc growth during the third instar.

In nature, avalanches are ubiquitous. From earthquakes to ferromagnets, large scale structural rearrangements arise, whereby a small perturbation leads to a pronounced collective response [8,9]. Systems characterized by such avalanche instabilities exhibit self-organized criticality (SOC) [10]. A main result is that SOC systems give rise to power law scaling signatures in observables, and by fitting to power laws, universal exponents can be obtained. These inferred exponents can be used to identify the strength of correlations in the system and the universality class of the process. Universality classes are groups of processes that share the same scaling properties. As an example, amorphous materials, which include glassy systems, exhibit avalanche behavior when exposed to strain [11,12]. A successful characterization of such avalanches utilizes the physics of self-organized criticality and phase transitions [11,13]. Recently, sheer stress induced avalanches were identified in the context of a vertex model, and both their universality class and material properties were mapped to amorphous materials [14]. Furthermore, signatures of criticality were identified *in vivo* for the *Drosophila* wing disc epithelium, suggesting that the nonlinear properties of collective processes in amorphous materials and the vertex model are at play in biological tissue growth [14].

Epithelial tissue growth and migration has been the subject of several recent *in vitro* studies. These generally branch into two categories, either focused on confined tissues [15–18], or on freely expanding tissues [19–22]. In the former category, processes that involve density induced motility transitions, front propagation, and spatial sensing are probed. In the latter category, processes involving mesenchymal-to-epithelial transition, contact inhibition, size dependence of growth and boundary formation are investigated. In both categories, cell motility and collective phenomena are a central theme. In the context of growth, a ubiquitous collective phenomenon, identified *in vitro* for both freely expanding and constrained growing cell tissues, is the formation of vortices by cell trajectories [18,20]. Similar to our observation of avalanches during growth, which is reported here, vortices are a density driven collective phenomenon [18]. Interestingly, the cell trajectories during avalanches also exhibit vortex signatures. However, the avalanches also involve a coordinated radial motion of cells not reported in [18,20]. In our study, the underlying model treats the tissue in a confluent state, as in epithelial tissues *in vivo*, where growth occurs in compartments separated by boundaries. Thus, it provides an appropriate framework, linking the phenomena probed with what takes place *in vivo*.

Biophysical properties, such as response to mechanical forces, impact cellular processes during growth phases in development [23]. Epithelial cells have been shown to regulate their shape and division rate by responding to mechanical force stimuli [15,24–26]. Furthermore, control of the cell division plane via cell-shape and mechanical considerations has been experimentally detected in animal and plant epithelial cells [24,27]. Numerical simulations of epithelial tissue growth, which include mechanosensing and division plane control, have shown to generate improved epithelial mosaics that represent *in vivo* structures more closely [28,29]. Single cell resolution computational models are a valuable tool to model growth since each cell can respond to its distinct state and incorporate phenomena at the single cell level [30,31].

Here, we use a vertex model [32] to simulate the spatial dynamics of confluent epithelial tissues with single cell resolution. This type of epithelial tissue computational modeling treats cells as polygons and uses energy considerations to track the spatial arrangement of cells, vertices, and edges. It has been successful in simulating apoptosis, mitosis, and cell shape changes [33,34]. In the vertex models, each edge, perimeter, and area of the cells contribute to the energy and affect the structure of the tissue. There are two main categories of dynamics implemented in vertex models. The first kind is the active vertex model, which explicitly updates the structure using the equations of motion for each cell or vertex in the overdamped regime [35]. The other kind is the quasistatic vertex model, which assumes that relaxation occurs in very short time scales and drives the system to the first accessible local energy minimum using gradient descent [32]. The local energy minima correspond to mechanically stable configurations, as the negative of the gradient of the energy corresponds to the forces on the vertices. These tissue models have been verified in Drosophila development with experimental data and laser aberration experiments, and have been recently successfully implemented in describing complex phenomena such as epithelial tissue folding and growth [32,35,36].

Epithelial tissues, across metazoa, have characteristic cellular packing geometries and distributions captured by the vertex model. One characteristic of epithelial geometry is formulated in Lewis’ law, which states that the average cell size for cells with n neighbors, $\bar{A_{n}}$, is proportional to the number of neighbors [37]. This characteristic distribution has been found in a variety of metazoan tissues [32,38–40]. Another characteristic signature prevalent across growing epithelial tissues is the distribution of cell sides [28,29,32,41]. The cells are predominantly hexagonal, with pentagons and heptagons following suit. These experimentally observed cellular distributions have been reproduced by models that combine single cell geometrical configurations and proliferation [28,30,32]

To investigate epithelial tissue growth in the context of an *in vivo* system, we simulated epithelial growth in the *Drosophila* eye disc during the third instar. The *Drosophila* eye disc is part of the eye-antennal disc, which begins with approximately 70 founder cells and develops to a final size of approximately 44,000 cells [42]. During the first half of the second instar, the eye-antennal disc grows via rapid cell proliferation, and at the late second instar, it segregates into the retinal progenitor and the antennal/head epidermal fields [43]. Photoreceptor development initiates at the beginning of the third instar, on an elliptical epithelial sheet, the retinal progenitor, composed of undifferentiated cells. The size of the retinal progenitor starts at approximately 10,000 *μm*^2^ [44]. At that stage, a visible indentation of the tissue, termed the morphogenetic furrow (MF), begins propagating linearly from the posterior to the anterior of the eye disc, leaving behind differentiating photoreceptors [43,45–47]. Cells within the MF region are constricted, with their apical surface reduced by a factor of about 15, from 9 *μm*^2^ ahead of the furrow to 0.6 *μm*^2^ [47]. There are two mitotic waves, the first mitotic wave occurs anterior to the MF, where cells differentiate asynchronously, and the second mitotic wave affects undifferentiated cells between photoreceptor cells behind the MF [47,48]. The first mitotic wave produces the majority of cells of the final eye disc and takes place ahead of the MF, whereas the second mitotic wave ensures that there are sufficient cells for proper ommatidial formation [42]. The second mitotic wave is more controlled, takes place posterior to the MF, and occurs in a synchronized and organized manner [49]. The contribution to growth of the second mitotic is negligible compared to the first mitotic wave and will not be included in the model [44].

The growth dynamics of the third instar *Drosophila* eye disc have been described quantitatively. The growth rate over time has been determined by analyzing experimental tissue size data over different developmental time points [44]. Both exponential decay and inverse area functional forms fit the data exceptionally well [44]. Furthermore, the area rule has been the focus of a subsequent study, which identifies a diluting and decaying transcript, cytokine unpaired, as a possible candidate for cell growth regulation [50]. Thus, functional forms and coefficients that describe the time dependent eye imaginal disc growth rate quantitatively are available and were used to simulate the process.

In order to simulate the propagation of the MF, cellular signaling was incorporated on top of the vertex model. *In vivo*, the MF is a result of signal transduction of multiple morphogens with multiple roles. The primary signal that is associated as the initiator and driver of the MF is hedgehog (Hh) [43,46,51–53], a morphogen that is sourced from differentiating neural cells posterior to the MF. Hh diffuses in the epithelial tissue up to and including the constricted cells in the morphogenetic furrow [46]. The propagation of the MF is mediated by Decapentaplegic and regulated by Hemothorax, Hairy and extra macrochaetae [46,54–56]. In the model herein, a simplified gene regulatory network was used, see [57] for a model with a detailed treatment of the gene regulatory network. The propagation of the MF was simulated by introducing an effective morphogen that induces its own expression while diffusing from posterior to anterior (see Computational Methods for details).

Few studies have so far been published that combine signal transduction processes, transcriptional responses, and vertex models [31,58–61]. In the model by Wartlick et al, the authors were able to reconstruct the observed growth of the wing disc via an integrated signal transduction and the vertex model by Farhadifar et al [61]. Schilling et al. developed a model for the production, diffusion, and sensing of Hh in the Drosophila wing primordium and explained how anterior/posterior cell sorting can be explained by a homotypic boundary model [59]. We will use a similar approach, i.e., a finite-volume method defined by a tessellation of the centroids of the cells. Notably, an alternative approach has been introduced utilizing the Lagrangian–Eulerian (ALE) formulation and finite element method to describe morphogen transport within a tissue coupled to a vertex model [60]. Furthermore, an earlier study was able to model wing disc growth by considering complex mechanical feedback, with cell constriction affecting the cell cycle and a biased angle of mitosis [30]. Finally, a report on the zebrafish retina was able to model highly ordered packing via coupling planar cell polarity proteins with edge tension [31,33]. The eye disc model developed here belongs to the class of models that couple signaling and vertex models. In the context of the eye disc, the model is novel in its treatment of the epithelial structure and a natural extension of the viscous fluid-based treatment of drosophila eye disc growth seen in [57].

The results section is structured as follows. First, the simple case of a circular epithelial tissue with a time independent growth rate is examined. In this part, the computational model is introduced, and avalanches are identified and analyzed within this simple setting. Second, the more intricate developmental process of *Drosophila* eye disc growth is studied, using a realistic computational model. Additional model components are introduced, and the onset of avalanches is investigated within this context. The division rate, propagation of the signaling wave and initial tissue shape and size are all calibrated using experimental data. The eye disc is a particularly interesting system, as there is coupling of mechanical deformations, from the coordinated apical constriction in the MF, and growth. This allows for avalanches to be identified in a simple model, which is straightforward to analyze, and in a more complex model, which incorporates more realistic features.

## Results

To understand a complex and novel phenomenon, such as the cellular avalanches identified here, it is advantageous to take a careful look at the simplest system possible. For this reason, we first analyze avalanches in the context of a circular epithelial sheet with time independent, spatially uniform growth. However, it is also important to study avalanches in the context of a biological system, using experimentally relevant parameters. Consequently, we build a model of *Drosophila* eye disc growth that allows us to make experimental predictions and to study the coupling of coordinated mechanical effects and growth. Notably, this is the first *Drosophila* eye disc growth model utilizing the vertex model.

### Generic uniform growth model

**Fig 1:**
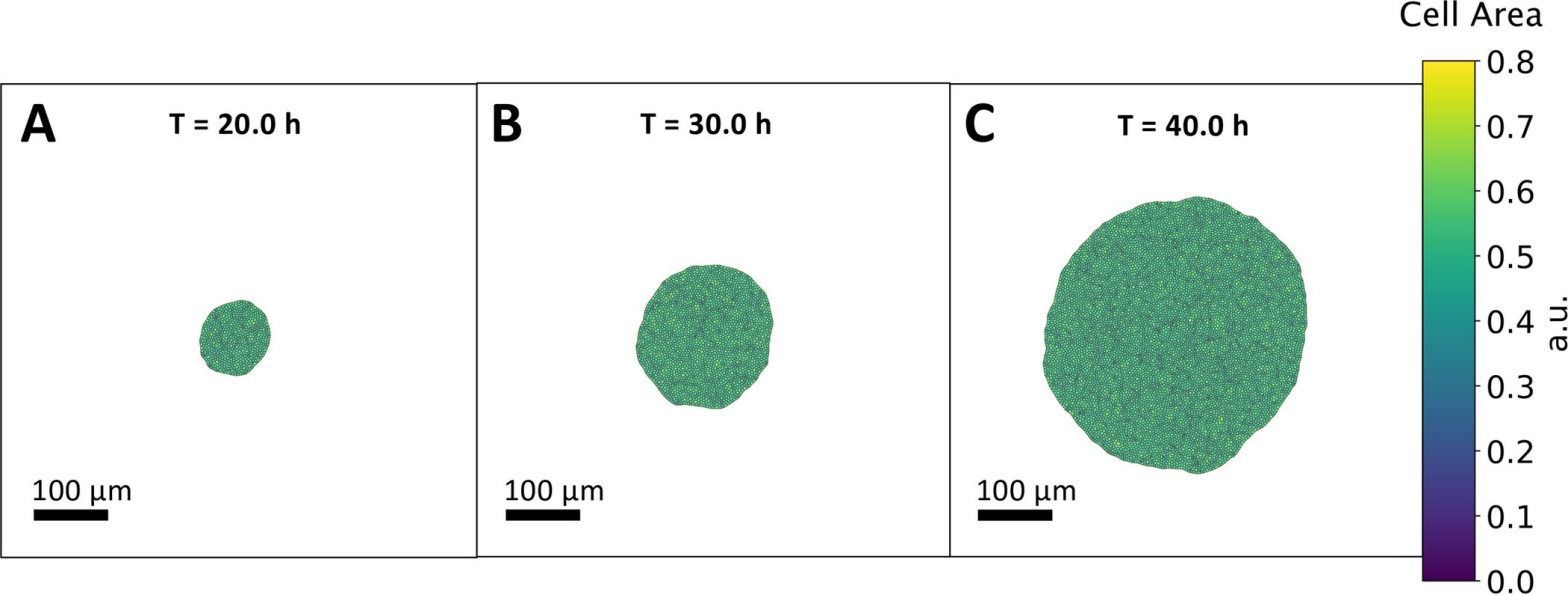
Uniform growth model. **A-C:** Successive frames of the tissue growth process at different time points, *T* = 20.0*h*, 30.0*h* and 40.0*h* respectively. The tissue starts at a small size composed of 50 cells and is simulated until it reaches 350 times its original size, allowing the analysis of different growth regimes. Cell division takes place at a constant doubling rate of ~6 hrs. The cells are color coded with respect to their area. The tissue retains a smooth boundary during growth and an approximately circular shape. In early times, the tissue grows continuously and exhibits discontinuities, or avalanches, at later times of ~40 hrs.

In order to study cellular avalanches during epithelial tissue growth, a vertex model is used, which is based on an energy functional that optimizes cell surface area, and minimizes edge tension as well as contractility of the cell perimeter. In our initial implementation, the tissue is initialized to a small circular size and is allowed to proliferate into empty space. The cell doubling rate is set to be the same for each cell and constant over time. In addition, the doubling rate is also set to weakly depend on cell area [29], approximating a type of cellular mechanosensing, with bigger than average cells having a higher division rate compared to smaller cells (see Computational Methods for details). The doubling rate is set at ~6 hrs, which corresponds to the maximal doubling rate observed in the second instar *Drosophila* eye disc. Changing the growth rate to a lower value within this implementation, would simply change lead to a nonlinear rescaling of the time axis. Contrary to the periodic boundary condition case, we discovered the addition of size dependent doubling rate was necessary for open boundary growth without significant cell death (S1 Fig). Without mechanosensing, the cells become unrealistically packed, there is significant apoptosis, and the increase of tissue size is suppressed. Importantly, avalanches were detected with or without the size dependent doubling rate. Furthermore, the tension at the tissue periphery is set to an increased value in order to enforce a smooth boundary. The approximately circular symmetry of this system allows for probing the radial profile of structural quantities. Three frames at different times of a simulation realization are shown in Fig 1A, 1B and 1C. In Fig 1C, the tissue increases significantly in size, as a constant cell division rate leads to exponential growth. A complete video of a simulation of this growth process can be found in S1 Video.

**Fig 2:**
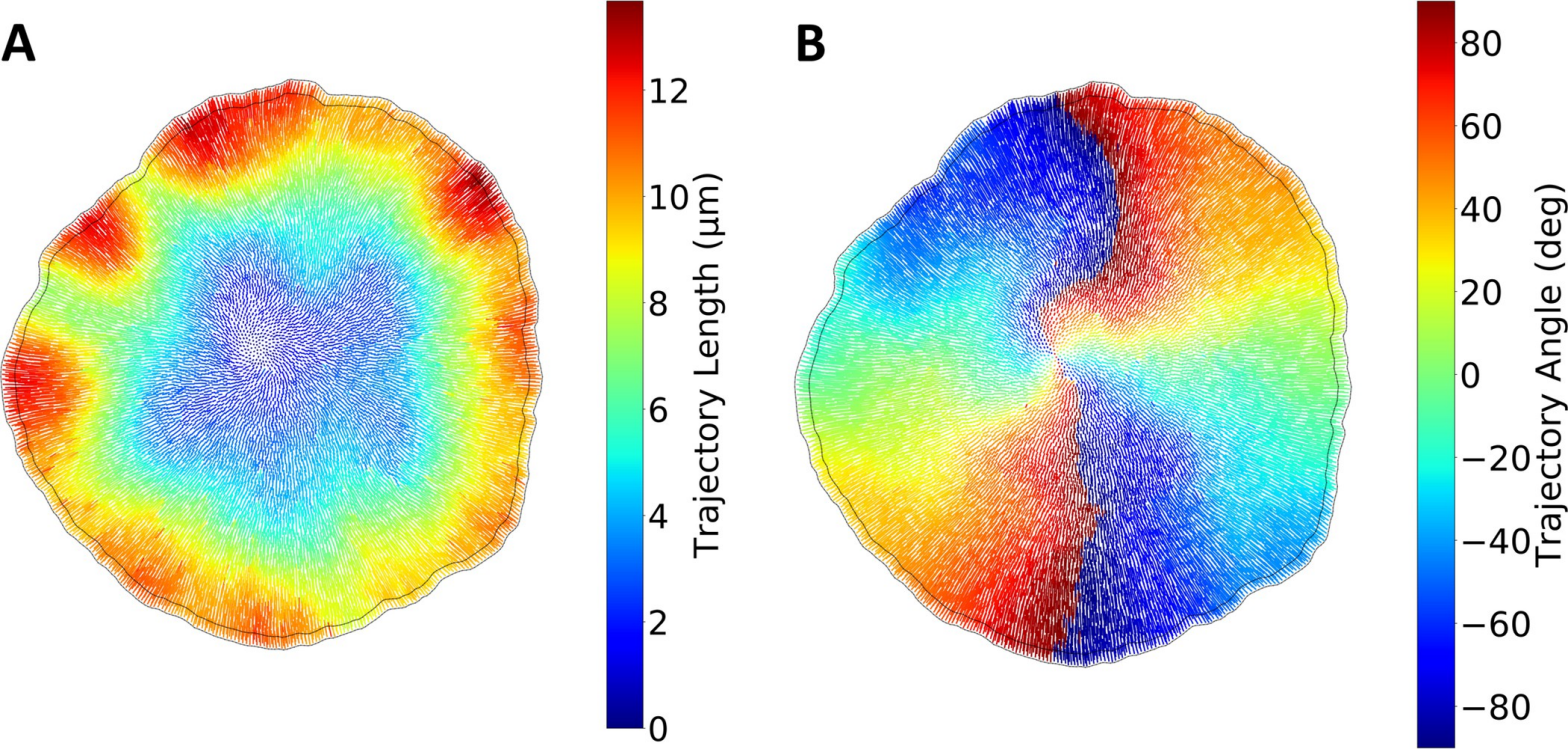
Single cell trajectory characteristics during avalanches. **A:** Cell trajectories during an avalanche, color coded with their respective length. The cells in the outer region displace the most, whereas the trajectories are shorter at the center of the tissue. The tissue boundary before and after the avalanche is outlined, visualizing the net expansion of the tissue. **B:** Cell trajectories during an avalanche, color coded with their respective angle relative to the x-axis, as defined by the inverse tangent function. On the outer region of the tissue, the trajectories are approximately radial. The vorticity is evident towards the center of the tissue, where the trajectory angles start to change.

Next, we probe aspects of the collective cell motility during a characteristic avalanche. In Fig 2A, the cell trajectories during an avalanche event are visualized. During the avalanche, the cells move collectively, with displacement increasing in a radial fashion. Interestingly, the trajectories of the cells also have a significant tangential component. The tangential component is visualized in Fig 2B by color coding the trajectories with their respective angles. Trajectories of cells in the center of the tissue have a significant tangential component, contrary to the radial direction of trajectories on the periphery. This signature indicates the presence of a cellular vortex. Interestingly, tissue scale vortex formation has been experimentally observed in growing epithelial monolayers [20]. In addition, the radial profile of the vortex, formed during an avalanche, is consistent with the profile reported experimentally [20]. For insights on T1 transitions during the growth process and the cell trajectories between the onset of avalanches, see S2 Fig.

**Fig 3:**
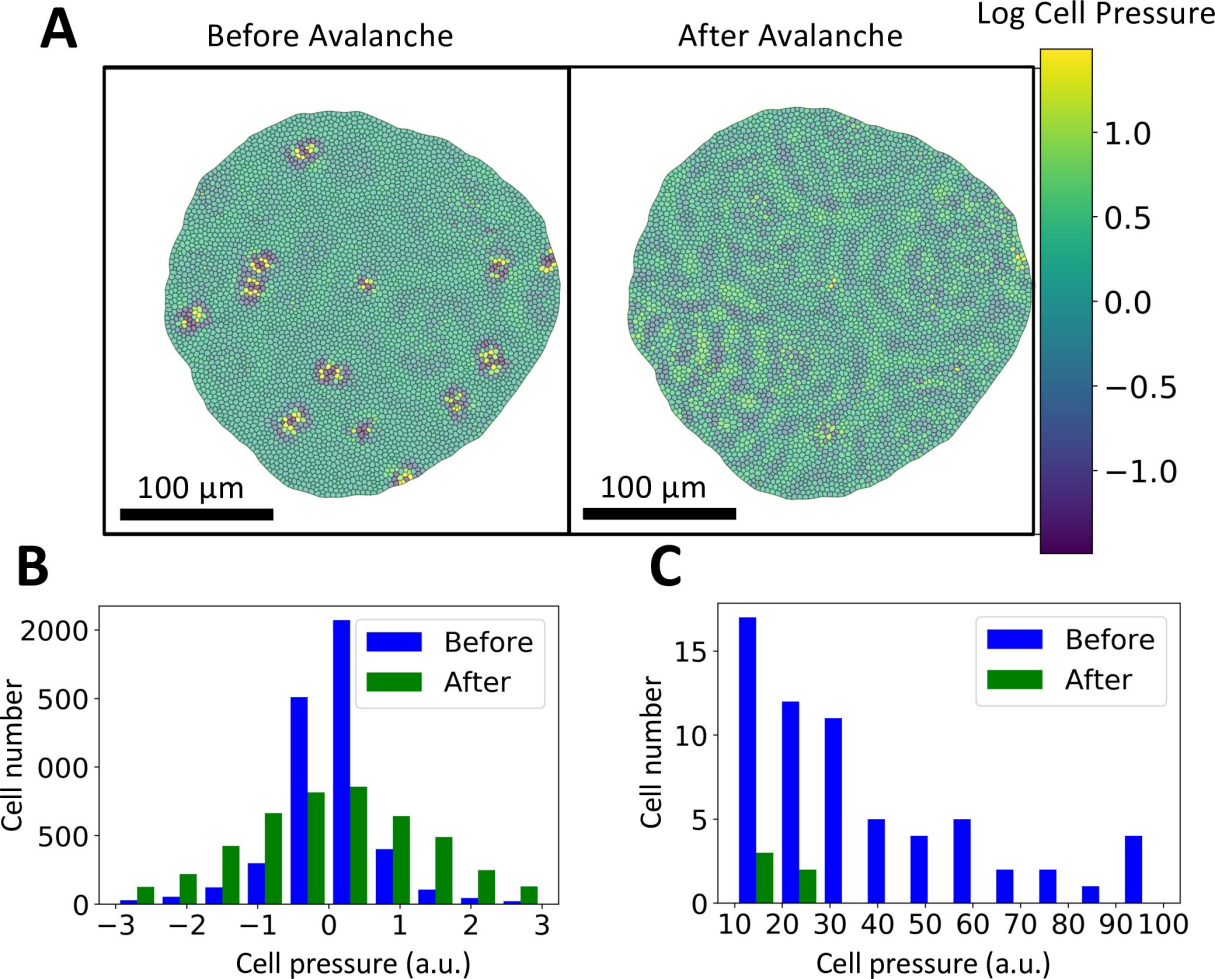
Cellular pressure characteristics during avalanches. **A:** Cellular pressure profile of the tissue before and after an avalanche, color coded with respect to magnitude. Before the avalanche, high and low pressure regions are localized. After the avalanche, the pressure redistributes across the tissue. **B:** Frequency histogram of cellular pressure before and after an avalanche. The histogram captures that the majority of cells have low pressure before the avalanche. Furthermore, the widening of the histogram captures the redistribution of pressure after the avalanche. **C:** Frequency histogram of cellular pressure before and after an avalanche for high pressure ranges. This histogram captures that, before the avalanche, there are hundreds of outlier cells with very high pressure which are not present after the avalanche.

To further understand the physical or mechanical origin and avalanches, we investigate cellular pressure profiles before and after the onset of an avalanche. To this end, the cellular pressure profiles and distributions before and after a characteristic avalanche are shown in Fig 3 (see Computational Methods for details). In Fig 3A, we visualize the pressure of each cell in the tissue before and after the onset of an avalanche. Furthermore, in Fig 3B and 3C we plot the pressure distributions over different ranges. Before the avalanche, the majority of cells have baseline pressure profiles, along with localized pockets with high and low pressure cells (Fig 3A and 3B). Upon closer examination of the pressure foci, we observe a propensity of high pressure cells being surrounded by low pressure cells and vice versa. After the avalanche, the pressure redistributes across the tissue, forming long distance pressure ripples (Fig 3A and 3B). Although the pressure distribution after the onset of an avalanche widens (Fig 3B), the pressure distribution before the avalanche includes a high number of outlier cells with very high pressure, which are absent after the avalanche (Fig 3C). Therefore, avalanches are accompanied by the redistribution of pressure from localized high and low pressure pockets to the entirety of the tissue.

**Fig 4:**
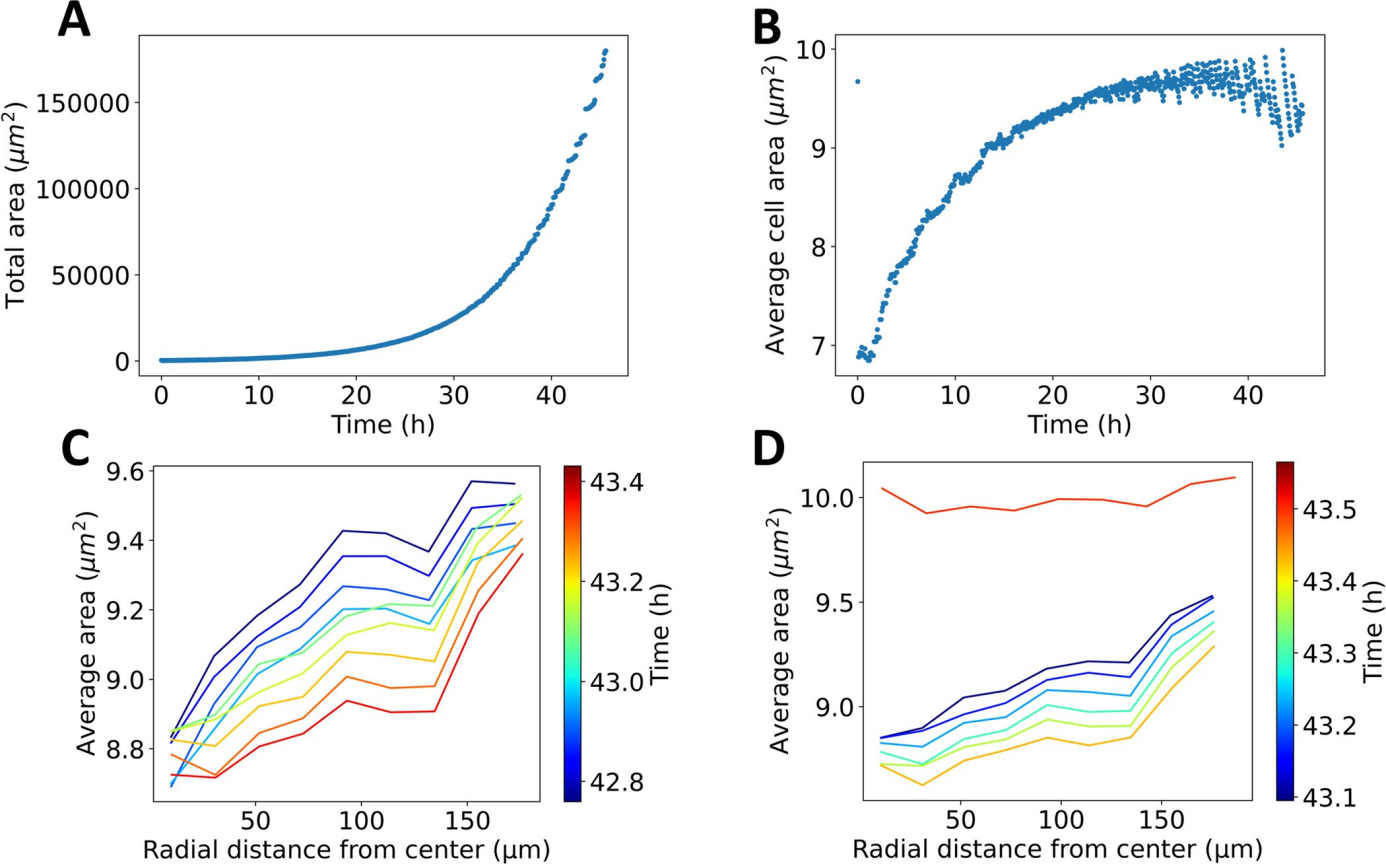
Uniform growth model observables. **A:** Total tissue area versus time. The tissue is initiated as a small size circle, and the cells undergo mitosis with a constant doubling rate. At early times, the tissue follows predominantly continuous growth. After ~30 h, the tissue displays growth predominately with discontinuities. **B:** Average cell area versus time. The discontinuities in average cell area become more pronounced and regular at ~30 h. **C-D:** Avalanches in the uniform growth model. **C:** Average cell area versus radial distance from the tissue center at consecutive time points. The spatial signature of the average cell area before an avalanche occurs is depicted. As cells divide, the average cell area decreases globally, with higher packing from the center to the tissue boundaries. **D:** Average cell area versus radial distance from the tissue center at consecutive time points. The spatial signature of the average cell area is depicted shortly before and after an avalanche occurs. Before the avalanche, there is a positive gradient from the tissue center to the tissue boundaries for cell size. After the avalanche, the average area increases with a discontinuity. The magnitude of the gradient diminishes.

The time dependence of the evolving total area and average cell area in this realization is depicted in Fig 4A and 4B. Initially, the tissue grows at a predominantly continuous rate, as cells can easily rearrange within the tissue. As it becomes larger, at approximately 30h, more pronounced discontinuities in the area growth are detected. These avalanches correspond to large scale cell rearrangements. Due to the initial small size of the tissue, the increased boundary tension dominates the energy term, and the tissue begins its growth at a high cell packing state. As the tissue grows further, the average cell area increases, as the boundary tension energy becomes negligible compared to the bulk energy.

The radial profile of the average cell area is probed to understand the onset of avalanches. Before the avalanche, the cell density increases as cell division takes place (Fig 4C). The spatial signature suggests increased cell packing in the center of the tissue, which diminishes outwards. Right after the avalanche, the radial cell density increases discontinuously (Fig 4D), and the gradient of cell packing is less pronounced after the avalanche. This is consistent with the radial increase of trajectory length shown in Fig 2A.

**Fig 5:**
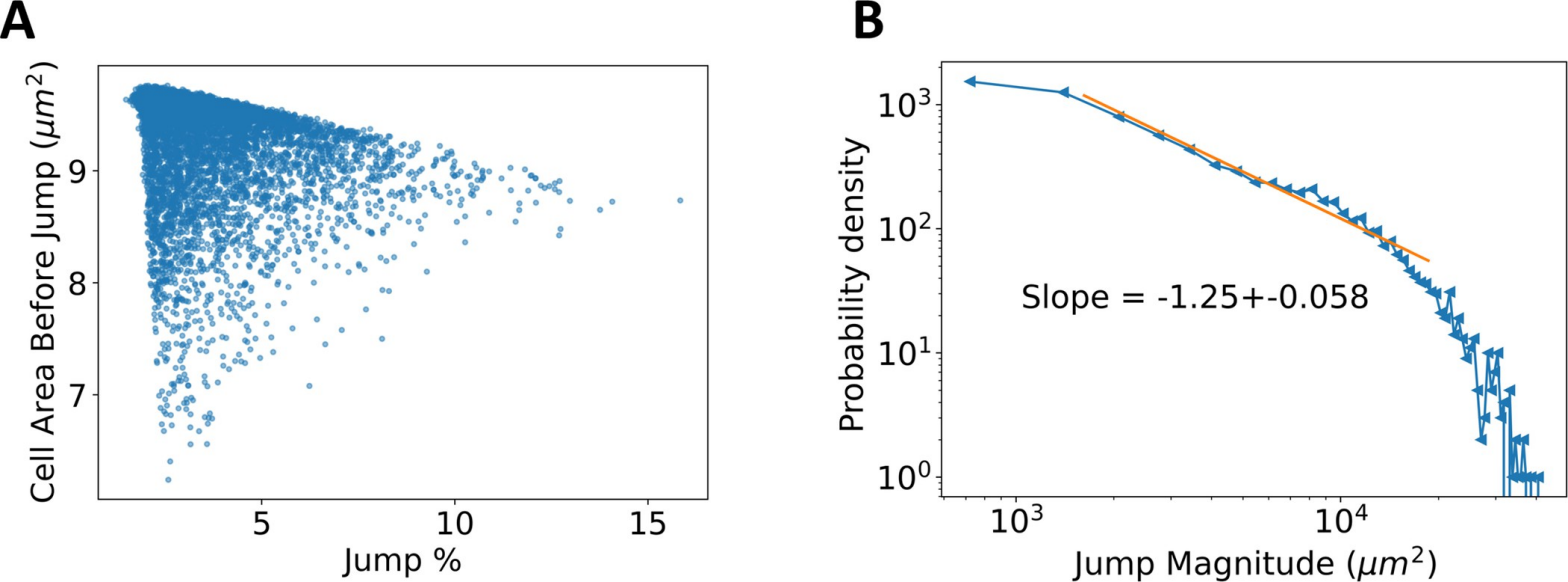
Avalanche Characteristics. **A:** Average cell area versus avalanche magnitude. The avalanche magnitude is quantified as the percent discontinuity in the total area. The more tightly packed the epithelium, right before the onset of the avalanche, the larger the total area discontinuity. **B:** Log-log plot of probability density of avalanche magnitudes. The functional form supports the presence of an underlying power law distribution, with a fit shown by the orange line. The exponent found is *τ* = 1.25±0.06. Such power law distributions are a universal characteristic of avalanches found in nature.

Based on these measured quantities, we can address what causes the avalanches and what controls their magnitude. To this end, we repeat 150 simulations of the process and isolate the avalanches. To do so, we skip the first 30 h of development and record an avalanche if a cell density discontinuity is equal to 0.1 *μm*^2^ or greater. To quantify the spatial inhomogeneity of cell packing, we use the standard deviation of the average area as a function of radial distance from the tissue center. Interestingly, Fig 5A shows that there is a strong correlation between the average cell area before the avalanche and the jump magnitude, with a spearman’s rank correlation coefficient of *ρ* = −0.46 (*p*<0.01). Furthermore, S3 Fig illustrates that there is weak correlation between spatial density inhomogeneity and jump magnitude. Thus, avalanche onset and strength are more sensitive to the global density of cells rather than to the details of their spatial distribution.

To understand whether the system exhibits self-organized criticality (SOC), the probability distribution of avalanche magnitudes is shown in Fig 5B. To provide more avalanches and solidify the fit, the growth model was run 150 times for an extended runtime, until the tissues reached 300,00 *μm*^2^. The logarithmic plot in Fig 5B suggests that the magnitudes are distributed according to a power law distribution, with an exponential cutoff, which is a ubiquitous property of SOC. The scaling exponent with its associated error is found to be consistent with *τ* = 1.27, in agreement with analogous exponents observed in sheer induced, stress avalanches in overdamped elastoplastic models [11,62]. More information about the fitting procedure and comparison with exponential fit is shown in S4 Fig. This result reinforces the notion that the vertex model and epithelial tissues share common material properties and universality classes with plastic amorphous systems [14]. To verify the presence of the power law signature, we varied different model parameters and repeated the scaling exponent analysis for two additional cases. Disabling T1 transitions and decreasing the mechanosensing amplitude did not cause the exponent *τ* to change, but rather yielded even better agreement with stress avalanches in overdamped elastoplastic models (S5 Fig).

### *Drosophila* eye disc growth model

In order to study the cellular avalanches in the context of a particular developmental process that is experimentally accessible, a more detailed model was developed. The *Drosophila eye disc* is also a valuable system to study due to the coupling between growth, signaling and coordinated mechanical deformations. In this version of the model, the cell doubling rate is set by experimental data, the tissue is initialized in an elliptical shape, and there is signaling of a single effective morphogen which drives the propagation of the morphogenetic furrow (MF) (see S1 Table and Computational Methods for details). The boundary tension is again set to the same increased value as the original uniform growth model to ensure a smooth boundary. Notably, the T1 length was set to a lower value than in the uniform growth model (S1 Table), with the aim to prevent cells from migrating when subjected to the stern apical constriction of the MF. A complete video of a simulation of this growth process can be found in S2 Video.

To calibrate the length scale in the model, the average area of a cell in the tissue was equated to the experimental average of apical area in the anterior region of the eye disc obtained from [44]. Furthermore, to set the time scale in the model, the speed of the MF was used. Experimentally, the MF propagates with a constant speed of approximately 3.4 *μm*/*hr* [44,57]. Having set the spatial scale, the time scale of the model was determined by equalizing the experimental speed of the MF to the respective simulated speed. Having set the spatial scale conversion, the initial conditions of the model were set to the experimental values for shape [57] and size [44] of the tissue at the initiation of MF propagation. Finally, having set the time scale, the growth rate of the anterior region Drosophila eye disc was measured [44,50] and matched by adjusting the average doubling rate of cells in the model. Details on the procedure and exact values and factors to convert from simulation units to physical units can be found in the Computational Methods section and S1 Table.

**Fig 6:**
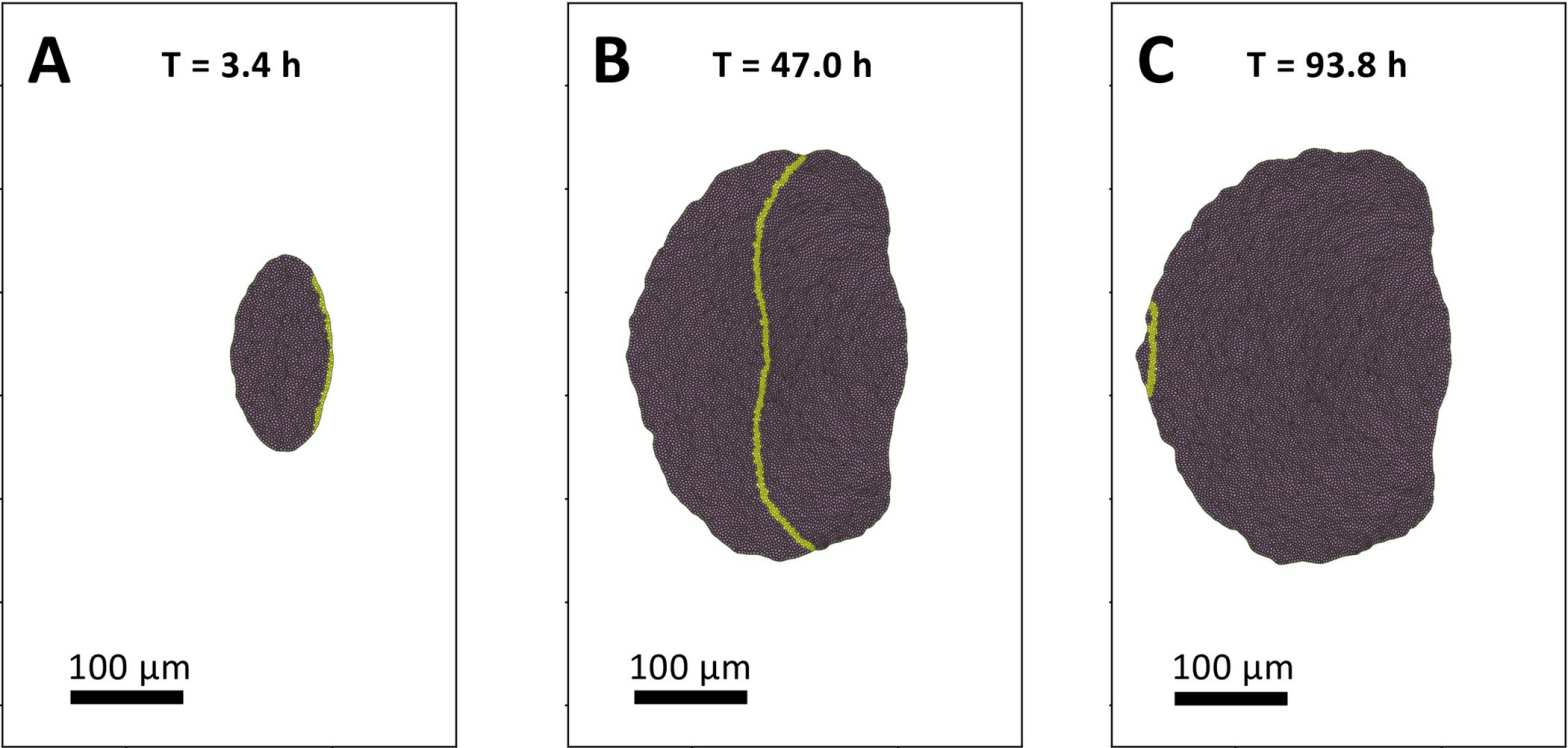
Drosophila eye disk tissue at different times of growth. **A-C:** Vertex model simulation of tissue growth, with cells in the MF highlighted in yellow. **A:** Time *T* = 3.4*h*; the MF is initialized at the posterior tissue boundary. **B:** Time *T* = 47.0*h*; the majority of cell divisions have taken place, while the MF has linearly propagated to the bulk of the tissue. **C:** The MF reaches the anterior boundary of the tissue, signaling the cell cycle arrest of all cells and termination of growth.

A visualization of the simulation of the growth process at three different time steps, initiation, middle, and end of growth are shown in Fig 6. Initially, the transcription factor is induced in the posterior boundary cells, leading to the initialization of the MF (Fig 6A). This will eventually cause the auto-activation of transcript production in neighboring cells, and will lead to the initiation of the MF. At this time point, all the cells are dividing in the tissue. At the midpoint, the MF has traversed approximately halfway through the tissue (Fig 6B). Cells entering the MF immediately stop dividing and become immobile 2.5 h after the MF has passed, to simulate differentiation. At the midpoint, the growth rate has decreased by approximately 80%, and the majority of cell divisions have taken place. Finally, at the end point (Fig 6C), the growth process is complete, as the MF has propagated to the anterior tissue boundary, and all cells have arrested their cell cycle.

**Fig 7:**
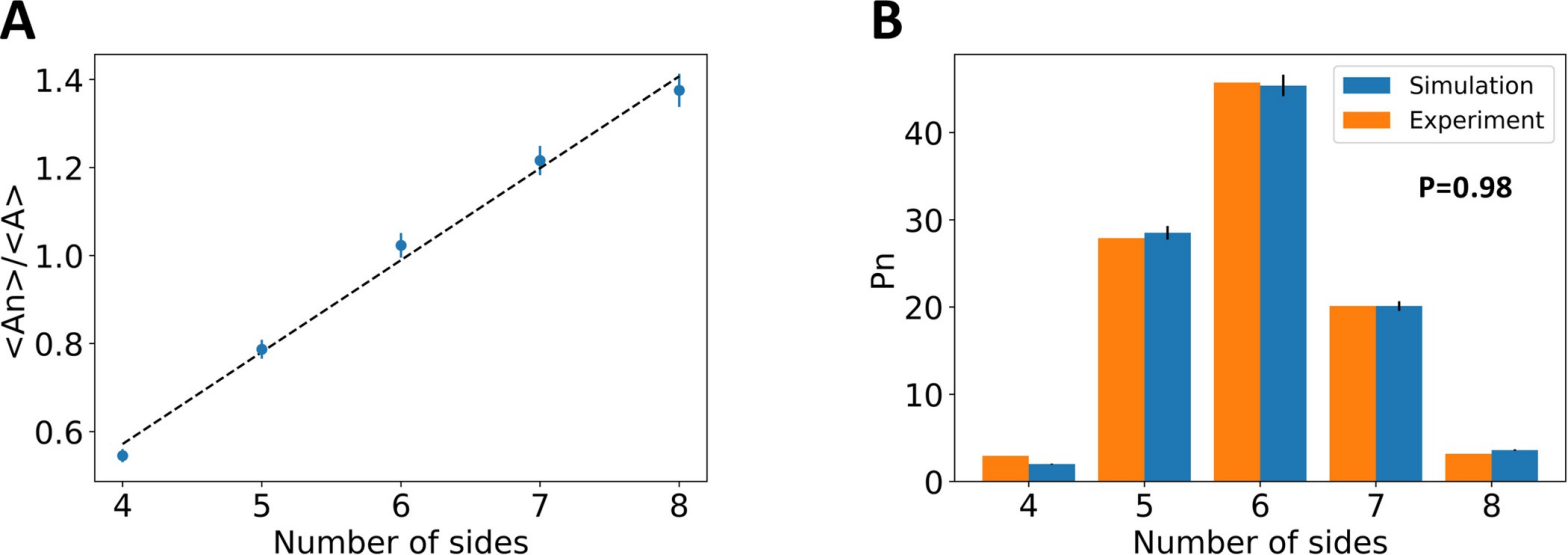
Quantifying epithelial packing during the simulated growth process. **A:** Average cell area per number of cell sides, divided by the mean area, is plotted versus cell number of sides. The values are obtained by combining data from all frames in the simulation. A linear relationship is observed, in accordance with Lewis’ law. **B:** Distribution of number of cell sides. The simulation values are obtained by combining data from all frames in the eye disc simulation. The experimental values were obtained from [41] and correspond to *Drosophila melanogaster* third instar larval wing disc.

During the growth process, the structural details of the cellular mosaic of the tissue were measured. To quantitatively describe the mosaic of the epithelial cells, average areas versus number of sides and the distribution of polygon classes were analyzed (Fig 7). The measured quantities match experimentally established, system independent, epithelial structure signatures. The emergent relationship between average cell size of a given polygon class, versus number of edges is linear (Fig 7A), in accordance with Lewis’s law. Furthermore, the distribution of cell polygon classes matches the corresponding distribution found *in vivo* (Fig 7B) with *P* = 0.98. Thus, the model gives rise to realistic epithelial packings.

**Fig 8:**
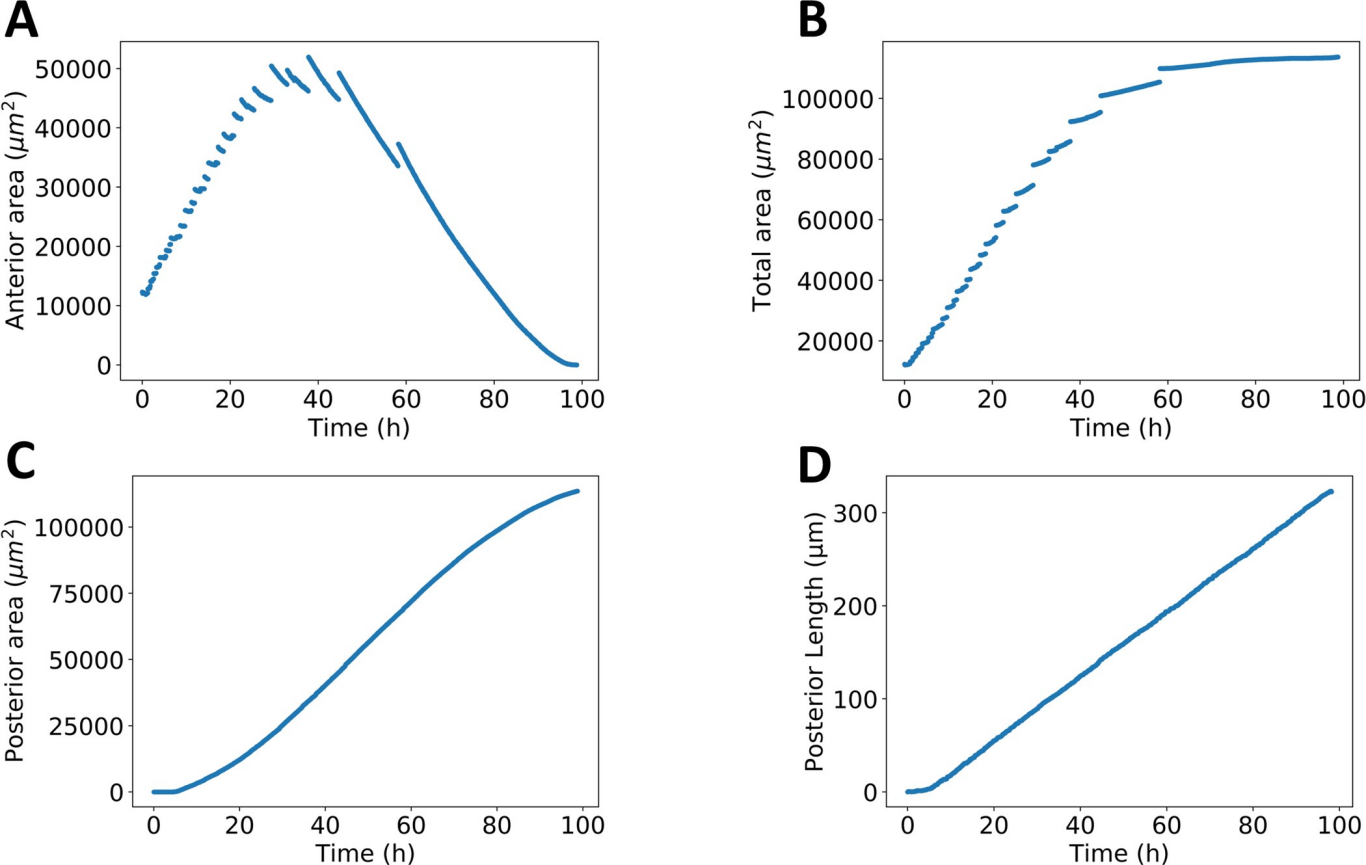
*Drosophila* eye disc growth dynamics. **A:** Time dependence of anterior area, defined as the portion of the tissue ahead of the MF. Cell division takes place only on the anterior portion of the eye disc. **B:** Time dependence of total area. The final value is in the same order of magnitude found in wild-type *Drosophila* strains. **C:** Time dependence of posterior area, defined as the portion of the tissue before the MF. Cell division is arrested in the posterior portion of the tissue. The posterior grows solely by cells exiting the propagating MF. **D:** Time dependence of the posterior length, defined as the distance of the posterior edge to the midpoint of the MF. Aside from initialization time, the posterior length increases linearly, with a slope of 3.4 *μm*/*hr*, in accordance with MF propagation experimental data.

Aspects of the tissue growth process can be captured by quantifying the temporal evolution of key observables. The anterior area depicted in Fig 8A initially grows, reaches a plateau and then decreases until it vanishes, as needed for growth termination. There are three effects contributing to the anterior area profile. Firstly, new cells are introduced due to cell mitosis. Second, since the MF separates the anterior from the posterior compartment, as the MF moves across the tissue, it turns anterior cells to posterior cells. Third, the rate of mitosis in the anterior decreases over time. The posterior area depicted in Fig 8C grows solely via the cells that exit the anterior area due to MF propagation. Initially, the eye disc is small, and the MF passes through a small number of cells. At the midpoint, the posterior area grows the fastest, as the MF is at the center of the tissue and passes through a large number of cells. Towards the end of the growth process, it tapers down, as it reaches the anterior edge. The total area depicted in Fig 8B is the sum of the anterior and posterior compartments. Finally, the posterior length depicted in Fig 8D, is shown to increase linearly over time. It is defined as the length from the posterior edge midpoint to the midpoint of the MF. Initially, there is a plateau, which corresponds to the initialization time needed for the MF to begin propagating. Casting aside the initialization time, the linear nature of the posterior length (*R*^2^ = 0.99) indicates that the posterior length indicates that the MF is propagating at a constant speed throughout the growth process. The value of the slope of the posterior length corresponds to the MF speed, which is 3.4 *μm*/*hr*, matching the experimental value.

**Fig 9:**
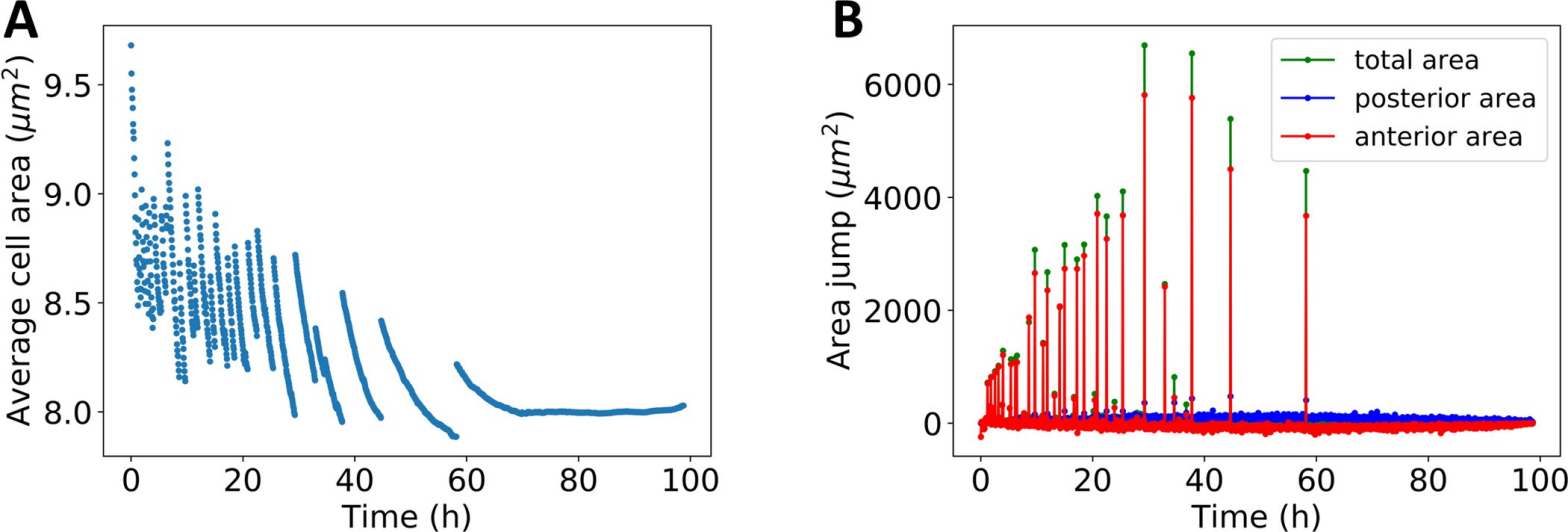
Avalanches in eye disc growth dynamics. **A:** Time dependence of the average cell area. The avalanches are captured by discontinuous transitions from high cell packing to low. **B:** Time dependence of tissue area jumps for total, anterior and posterior area, marked by green, red and blue respectively. The avalanches lead to tissue area discontinuities, with the majority occurring in the anterior.

Similar to the simpler model, the quantification of the growth process reveals discontinuities, i.e., avalanches, in the temporal evolution of the tissue area. This can be seen in the anterior area in Fig 8A, and consequently also in the total area (Fig 8B). To understand better what is taking place at the single cell level and across tissue compartments, the average cell area during the growth process is plotted in Fig 9A. In Fig 9B we visualize the tissue area avalanche magnitude for each tissue compartment. Comparing Fig 9A to 9B, one can see that each avalanche is accompanied by a discontinuity in the average cell area. Furthermore, the discontinuities take place after the average cell area decreases and always lead to an increase of the average cell area. Therefore, the avalanches are a consequence of increased cell packing in the eye disc that leads to a global rearrangement of cells. Also, the avalanches mainly take place in the anterior of the eye disc (Fig 9B).

**Fig 10:**
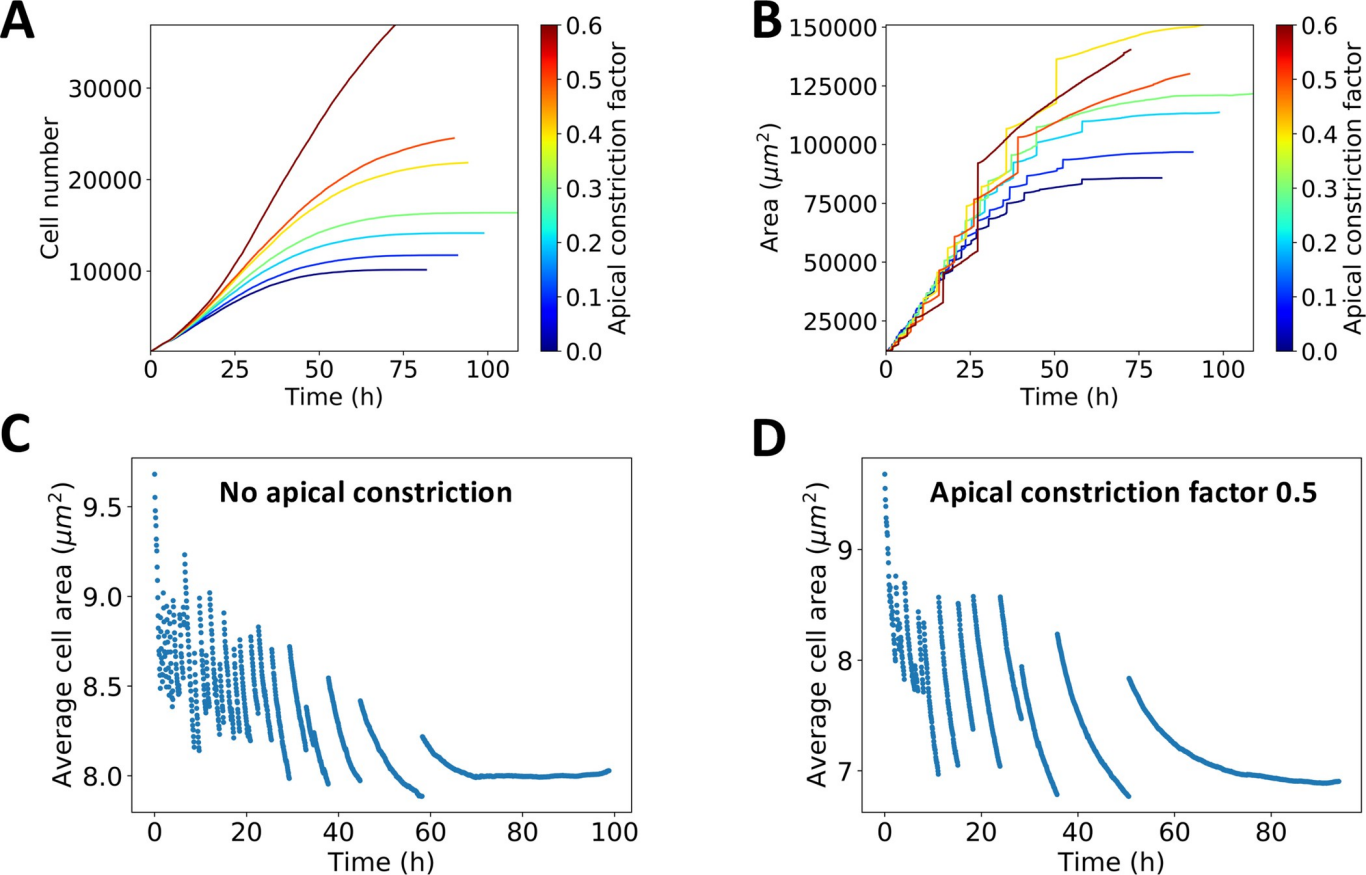
Avalanches depend on the magnitude of apical constriction in the MF. **A:** Time dependence of the cell number. The larger the apical constriction, the higher the cell number. **B:** Time dependence of the tissue area for different constriction strengths. The larger the apical constriction, the bigger the tissue area. **C-D:** Average area for no constriction and moderate constriction. The case of no apical constriction corresponds to more frequent avalanches that take place in higher densities. This highlights the observation that apical constriction in the MF makes avalanches harder to achieve and leads to higher cell packing.

Changing apical constriction affects the onset of the avalanches. In the model, we can tune the strength of apical constriction in the MF. In Fig 10, the MF cell constriction is incrementally increased from no constriction to high constriction, by changing the preferred area of cells in the MF. We define the apical constriction factor as the percent decrease in the preferred cell area of cells in the MF. Fig 10A shows the effect of the constriction strength on the cell number, with higher constriction leading to significantly larger cell numbers. On the other hand, Fig 10B shows total area, which reflects that eye discs are larger when the apical constriction in the MF is increased. However, their relationship is not a scalar multiple, as average cell size is variable between different apical constriction strengths. This can be seen by inspection of two simulation examples (Fig 10C and 10D) of the average cell size, one for no apical constriction and one for strong apical constriction in the MF. When the apical constriction is increased, the avalanches become less frequent and the average cell area before they take place is decreased. Thus, apical constriction makes it harder, in the sense that cells reach a higher packing state, for global cell rearrangements take place. Finally, since the reaction-diffusion equation is discretized on the lattice of the cells, independent of packing, a decreased cell size leads to a decrease in the MF speed (S6 Fig). Since the growth rate decays based on the posterior length and thus the MF speed, apical constriction makes the growth rate decay slower.

### Model predictions

The model predicts that proliferating epithelial tissues, with short relaxation time scale dynamics, exhibit discontinuous spurs of growth. The onset and strength of the discontinuities, which we term avalanches, are positively correlated with increased cellular packing. Thus, the model predicts that the average cellular area depends strongly on whether the tissue growth is before or after an avalanche. Furthermore, the model predicts that, prior to avalanches, localized high cellular pressure regions are formed, which redistribute across the tissue after the avalanche by forming pressure ripples. Lastly, the model predicts the formation of tissue scale vortices during growth, in agreement with experiments [20].

In the context of the *Drosophila* eye disc development, with the assumption that the dynamics fall into a regime of short relaxation time scales, we expect that the average cell density will exhibit considerable variation during growth. Since avalanches take place less frequently at later times during tissue growth, the variations should be less intense at later developmental times, consistent with a more densely packed eye disc. In addition, the stochastic onset and strength of avalanches suggests that cellular density should vary between eye discs at the completion of eye disc growth. Increase of the apical constriction in the MF makes avalanches harder to attain, with cells needing to be more packed. Thus, mutant eye discs with lower apical constriction should, on average, exhibit a larger average anterior cell size compared to the wild-type.

## Discussion

To date, most insights regarding the material properties of epithelial tissue have been based on a viscoelastic description. Namely, epithelial tissues have been shown to give rise to both solid and liquid material properties. In addition, numerous cellular properties have been identified that coordinate cell proliferation and regulate the mechanical properties of cells. When the tissue is small, we find that the relaxation processes available to the cells are sufficient to redistribute the proliferative stress. In this regime, the tissue retains its viscoelastic properties and grows in a continuous fashion. When the tissue becomes larger, proliferative stress builds up within the tissue and eventually leads to a discontinuous increase in area. Thus, the avalanches presented herein constitute a collective phenomenon whereby the tissue regains its fluidity and is able to explore the energy landscape and reach an energetically favorable state. This is an instance of non-Newtonian behavior, as an increasingly stiff tissue promotes fluid-like avalanche deformations.

Our vertex model implementation predicts that, as tissues grow, the growth process is dominated by avalanches of cellular redistribution. However, do epithelial tissues exhibit avalanches *in vivo* under proliferation stress? And which biological and biophysical processes control the onset and magnitude of the avalanches? Addressing these issues is imperative for developing realistic integrated biophysical computational models that accurately describe developmental processes. Especially, the interplay between growth and cellular signaling requires a valid description of the underlying cellular mosaic and its dynamics.

The discovery of avalanches highlights a central challenge that proliferating epithelial tissues have to overcome. When cells proliferate, especially in large epithelial sheets, structural stress is bound to build up. As a response, cells rearrange themselves and undergo topological transitions to relieve the accumulated stress. In addition, when stress builds up, cells are known to react to mechanical stimuli by regulating their mechanical properties and mitosis. However, what happens if the standard relaxation processes are not enough to release proliferative stress and it starts to accumulate?

The computational findings presented herein suggest that, if proliferative stress accumulates in an epithelium, it will eventually lead to a collective rearrangement, i.e., avalanches. Without these avalanches, the tissue would not be able to release the accumulated proliferative stress and would either lead to an overcrowded epithelium, widespread cellular apoptosis, delamination, and termination of growth. Therefore, the avalanches constitute a global mechanism that allows the redistribution and release of proliferative stress.

Similar to the signatures identified in our model simulation of avalanche dynamics, time resolved evidence of cell area oscillations have been reported in expanding epithelia [16]. However, so far, experiments that involve freely expanding cell cultures have not yet reported this phenomenon. One of the reasons may be the boundary conditions: in freely expanding epithelia, the boundary expands by forming protrusions with cells, often losing confluence. This is in contrast with the simulated system, where the tissue retains its confluency and smooth boundary. Given this inconsistency with previous in vitro studies of freely expanding proliferating epithelia, we propose that avalanches would be present if this difference was addressed. Specifically, growing the epithelia in a highly adhesive medium would allow boundary cells to maintain confluency throughout the growth process. Even if avalanches are not identified, mutations on main mechanotransduction pathways could lead to accumulation of proliferative stress and the prevalence of avalanche dynamics.

The computational model treats the tissue dynamics within a quasistatic paradigm. That is, it is assumed that the energy relaxation time scale is very short. Changing the solver from gradient descent to a simple overdamped dynamics solver, suppresses the onset of avalanches for a large range of viscosity magnitudes (S8 Fig). Interestingly, the implementation of the viscous solver does not converge to the quasistatic solver, even for low viscosity values. This is to be expected, since the quasistatic solver utilizes a second-order optimization algorithm that converges at local energy minima. The step size and number in the viscous solver would need to be further tuned to recover the output of the quasistatic algorithm. These considerations highlight a central question: which solver, and in which biological context, is best at capturing epithelial tissue dynamics? Although the viscous solver is based on physical considerations, the fluctuations and adaptive responses that take place in a tissue might make it able to rapidly explore the energy landscape and find a local minimum faster than the viscous solver

The magnitude of viscosity, cell properties and the nature of cellular fluctuations control the strength and prevalence of avalanches. For instance, when the cellular edge tension is incrementally decreased and the cellular network transitions from hexagonal to a soft network as shown in [32], we find that the avalanches no longer take place (S9 Fig). In general, when the relaxation time scale increases, we expect that the avalanches are smoothed out until they cannot be resolved. *In vivo*, the relaxation time depends on details of the cells, such as cell cycle progression, expression profiles of cadherins and acto-myosin activity. Therefore, the choice of dynamical update rules has profound and fundamental effects on collective phenomena and growth processes, stressing the importance of experimentally quantifying fluctuations and relaxation timescales [63,64]. Our computational study illustrates the intricacy of growth processes, their dependence on the dynamics and emergent phenomena when coupling signaling and mechanical responses.

### Computational methods

The complete code, parameters, and instructions on how to run it are provided in the public github repository https://github.com/gcourcou/ChemoMechScript.git.

### Spatial structure

There are many manifestations of vertex models. Here, we adapt a model introduced by Farhadifar et al, which has been successful in emulating the growth of *Drosophila* wing disk epithelia. In this model, the tissue is assumed to evolve quasistatically, i.e., the tissue re-arranges instantaneously to the nearest local minimum via gradient descent [32]. This approach originally uses a 2D description of the tissue, but it can be generalized and applied in 3D [36]. The energy function considers cell elasticity, actin myosin bundles, and adhesion molecules. Mathematically, it contains an elastic term for area, tensions for each edge and contractility for the perimeter,

$$E(\vec{R})=\underset{\alpha }{\sum }\frac{K_{\alpha }}{2}{\left(A_{\alpha }-A_{\alpha }^{\left(0\right)}\right)}^{2}+\underset{<i,j>}{\sum }\Lambda_{ij}l_{ij}+\underset{\alpha }{\sum }\frac{\Gamma }{2}L_{\alpha }^{2},$$

where α is the cell index, *K*_*α*_ is the elastic coefficient for compressibility, *A*_*α*_ is the area and *A*_*α*_^0^ the preferred area, <i,j> is the summation over all edges, *Λ*_*ij*_ is the edge tension and similarly *Γ*_*α*_ is the contractility of the perimeter L_*α*_. The above energy expression can be renormalized by multiplying by $\left(K\;{A^{\left(0\right)}}^{2}\right)$ to obtain the following expression:

Determining the epithelial tissue structure in a given conformation of cells is more intricate than sole gradient descent energy minimization. Cellular $E^{\prime }(\vec{R})=\sum_{\alpha }\frac{1}{2}{\left(A_{\alpha }^{\prime }-A_{\alpha }^{\prime (0)}\right)}^{2}+\sum_{<i,j>}\bar{\Lambda_{ij}}l_{ij}^{\prime }+\sum_{\alpha }\frac{\bar{\Gamma }}{2}L_{\alpha }^{\prime 2}$ structures undergo topological transitions, if they further minimize the energy of the configuration. The possible transitions have been addressed in the context of foam physics [65]. For instance, cells can undergo neighbor exchange via a T1 transition [32,34]. Cells do not have a fixed number of edges, and if it is energetically favorable, new edges can form via T3 transitions [32,36]. Contrary, cells that shrink undergo inverse T1 transitions, which reduce their number of edges. When a cell obtains three edges or reaches a critical apoptotic size, it can be removed from the structure via a T2 transition [32,65]. These transitions conserve the topology of the tissue while implementing energy minimization. The coding environment for the spatial structure was taken from the tyssue repository on github. The tyssue library (DOI 10.5281/zenodo.4475147) is a product of code refactoring originally from [36].

Neglecting the attachment of the eye imaginal disc to the antenna disc and the peripodial membrane [43], we approximate the eye disc to be suspended and with open boundary conditions. Due to the asymmetry of the growth process, as the MF linearly propagates, one cannot implement periodic boundary conditions, as it is often done in other systems [61]. To approximate the effect of boundary cells, increased tension was implemented at the cell edges along the boundary. The boundary tension parameters were chosen such that a circular tissue was energetically favorable (S7A Fig). The increased boundary tension did not lead to significant changes in the growth process (S7B Fig). This is motivated by the established differential adhesion on the tissue boundary layer, often thought as creating an effective surface tension [66,67].

To simulate the structural component of the morphogenetic furrow, a simplistic approach was taken. After determining the cells that make up the MF, their preferred area was instantly set to a decreased value. We term apical constriction factor the percent decrease in preferred cell area of cells in the MF. Furthermore, the preferred area for cells that exit the MF was set back to the default value. In the case of the eye disc model, 2.5 hrs after the cells exhibit the MF, they are artificially made immotile by no longer considering their contribution to the energy term, to simulate the differentiating region.

### Pressure visualization

In the context of the vertex model, and as shown in [24], cellular pressure can be calculated using the following equation:

$$p_{c}=-\frac{1}{A_{c}}\underset{i\in c}{\sum }\frac{\partial E_{c}}{\partial \vec{r_{i}}}(\vec{r_{i}}-{\vec{r}}_{c})$$

Where *p*_*c*_ is the pressure of cell c, *A*_*c*_ is the area of cell c, *E*_*c*_ is the energy of the tissue, ***r***_***i***_ represents the positions of the vertices of the cell, and *r*_*c*_ represents the center of the cell. To visualize pressure of cells on the tissue we used a logarithmic scale. Specifically, we used $\log_{10}\left(p_{c}^{\prime }+1\right)$ for positive pressure and ${-\log}_{10}\left(-p_{c}^{\prime }+1\right)$ for negative pressure values where *p*_*c*_′ = 1000 *p*_*c*_. The rescaling was done to provide contrast to the visualization, since the pressure values were small compared to 1. Note that all pressure results in the manuscript are reported in terms of *p*_*c*_′.

### Growth

In the vertex model, cell division can be achieved by directly introducing a new edge in a target cell. Some implementations include area growth, but it has been shown that area does not correlate with volume [29], so we use an alternate implementation. In this implementation, the new edge is chosen by drawing a line through the cell centroid with random orientation [30]. The two daughter cells inherit the concentration of the mother cell.

To simulate the cell cycle, a variable V, which corresponds to volume, is assigned to each cell [29]. This variable increases over time, and when it crosses a constant threshold, arbitrarily chosen to be 1, the cell divides. To model the mechanical feedback of the cells, also known as mechanosensing, the magnitude of increase over time includes an area dependent term [29]. This formulation has been shown to recreate epithelial cell topologies similar to the ones seen in experiments. Finally, to match the experimental cell doubling, the step increase of this is normalized by *t*_*norm*_(*k*), which depends on the growth rate k. The variable and its step increase are shown in the following equation:

$$\Delta V_{\alpha }(k)=V_{d}\cdot [\beta +\mu \frac{\left(A_{\alpha }-\bar{A}\right)}{A^{\left(0\right)}}]\cdot t_{\mathrm{norm}}(k)$$

In this equation *ΔV*_*α*_ is the volume contribution in a single iteration for cell α, *V*_*d*_ is the threshold volume for division, *β* is a constant growth contribution rate, *μ* determines the strength of mechanosensing contribution to growth and *t*_*norm*_ is a factor that matches the experimental growth rate. Finally, *A*_*α*_, $\bar{A},$ *A*^(0)^ represent the area of cell α, the average cell area in the tissue and the preferred area of the cell respectively. Note that the preferred area of a cell is a structure variable in the vertex model implementation.

The functional form of *t*_*norm*_ depends on the growth profile we want to use. For the exponential growth profile used to model the eye disc, $k=k_{0}e^{-\delta_{PL}L_{p}}$ [44,57], where *δ*_*PL*_ is a constant and *L*_*p*_ is the posterior length (used interchangeably with developmental time since *L*_*p*_ = *v*_*MF*_*t*). As an approximation, we assume that the target cell has an average area simplifying the equation to:

$$\Delta V_{\alpha }(k)=V_{d}\cdot \beta \cdot t_{\mathrm{norm}}(k)$$

Note that since cells start at *V*_*d*_/2 as a starting volume, they require *V*_*d*_/2 to divide. Now, *t*_*norm*_ can be derived from the equation:

$$t_{\mathrm{norm}}(k)=\frac{k_{0}e^{-\delta_{PL}L_{p}}}{2V_{d}\beta }$$

Where in the above, we assume that β and *k*_0_ are in units 1/*s*.

### Eye disc model: signaling

In order to describe the progression of the MF, a simplified approach was taken. The complete complex interactions of multiple morphogens and transcripts are not needed for this study. To that end, signaling was modeled by considering only a single effective transcription factor obeying an auto-activation regulatory loop. This transcription factor obeys the reaction diffusion equation shown below. The differential equation was discretized on the cell center lattice generated by the vertex model [61]. The lattice was approximated by a hexagonal packing and the appropriate discretization method was implemented [68].


$$\frac{d}{dt}c(r,t)=D_{c}\nabla^{2}c(r,t)+P_{c}\theta (c(r,t)-c_{0})-\lambda c(r,t)$$

In this equation, c is the concentration of the transcription factor *D*_*c*_ is the diffusion coefficient, *P*_*c*_ is the production rate and *λ* is the decay rate. The θ depicts a step function which becomes 1 when the concentration crosses *c*_0_. The emergent dynamics of this equation, after initializing a concentration or source, give rise to a non-dissipating linearly propagating concentration gradient. This is ideal for the eye disc, as the MF is known to propagate at a constant speed of 3.4*μm*/*hr*, as measured by the dorsal-ventral point of the MF [44]. Finally, cells that constitute the MF were defined as cells that have a concentration between a range, chosen to be 0.4 and 1.

Given the autoregulatory loop for MF propagation, an initial flux or concentration of the effective morphogen is necessary. *In vivo*, Hh diffuses through the posterior tissue boundary and initiates the signaling cascade necessary for MF propagation. To initialize the propagation of the MF in the eye disc simulation, we follow a similar approach with [57]. The posterior boundary cells obeying *x*(*t*)≤0.2 *L*_*AP*_, where *L*_*AP*_ is the total anterior to posterior length, received a boundary flux of the morphogen equal to 40% of the autoproduction rate *P*_*c*_. Varying this parameter simply changes the time scale of the initialization phase. Furthermore, the boundary flux was tapered down by a time dependent term *τ*(*t*) = 1−*θ*(*t*−10*hr*), such that the boundary flux ceases after 10 hrs.

### Model calibration and initialization

The specifications to initialize the eye disc size and shape were taken from [57]. An elliptical tissue was extracted from a previously grown epithelial tissue under periodic boundary conditions as in [29,32]. For the uniform growth model, a circular tissue with a radius of 11 μm was extracted from the same previously grown epithelial tissue. At the start of both uniform growth and eye disc simulations, cells are set to be at a random point of their cell cycle. That is done by setting the variable V, defined above, at a random value from 0.5 to 1. Note when *V* = 1, a cell divides and V is halved, making the minimum V be 0.5.

Next, we discuss how we set the spatial and temporal scales in the simulation. Firstly, the time and space scales used in the generic uniform growth model were taken from the eye disc. Given that the average cell size in the posterior portion of the eye disc is known to be 9 *μm*^2^ [47], obtaining the average cell area from a sample simulation and equating it to the experimental value provides the length scale. For an intermediate value of constriction, the MF propagation speed was measured. Then, it was equated to the experimentally derived value of 3.4 *μ*m/*hr* [44] to obtain the time scale. This allowed for the conversion of simulation time to developmental time and thus, the calibration of the growth rate. The apical constriction is known to cause the 9*μm*^2^ cells ahead of the furrow to 0.6 *μm*^2^ [47]. The two-dimensional vertex model cannot support such an intense constriction. To this end the apical constriction is varied and the corresponding emergent behaviors are investigated.

### Coupling signaling and tissue structure iterations

A custom script was developed for the eye disc model, which combined reaction diffusion based signaling and the vertex model. Firstly, the tyssue library was used for the vertex model and its dynamical updates. For signaling, which translates to ordinary differential equations under discretization, python’s scipy.integrate.solve_ivp() function was used [69]. The two aspects were updated sequentially. The differential equations were time-evolved for a constant interval of time. Then, the properties of the cells, a subset of which depend on the morphogen concentration, were updated. Afterwards, the tyssue solver was used to relax the system to the nearest energy minimum. Then, the configuration of the resulting structure was plugged in the differential equations, and they were time propagated. The time resolution of this transition between signaling evolution and mechanical update was increased until the macroscopic system observables were invariant. The mechanical structure and cell state were updated every 4 minutes of developmental time.

## Supporting information

S1 Table Key model parameters. Additional model parameters can be found in the github repository https://github.com/gcourcou/ChemoMechScript.git.
(XLSX)

S1 Video Video depicting a realization of the uniform growth model. (MOV)

S2 Video Video depicting a realization of the *Drosophila* eye disc growth model. (MOV)

S1 Data. Numerical data underlying graphics (XLSX)

S2 Data Output file of uniform growth simulation. This file contains all numerical outputs recorded during the simulation depicted in Fig 3.
(TXT)

S3 Data Output of eye disc growth simulation. This file contains all numerical outputs recorded during the simulation for the eye disc model depicted in Fig 7.
(TXT)

S1 Fig Mechanosensing analysis in the uniform growth model. **A:** Cell death during growth for different mechanosensing amplitudes. When mechanosensing is toggled off, at zero mechanosensing magnitude (μ), there is significant cell death due to proliferation stresses. When non-zero values of mechanosensing are used, cell death becomes negligible. **B:** Area versus time for different mechanosensing amplitudes. The mechanosensing amplitude significantly affects the growth process. Since this is exponential growth, small variations intensify exponentially. Close to *μ* = 0.04, which is the parameter used and is taken from [29], the area does not deviate when changing mechanosensing. Growth discontinuities or avalanches are present for all mechanosensing parameters. **C)** Division number versus time for variable mechanosensing magnitudes. When mechanosensing is turned off, the cells divide periodically since all daughter cells grow instantaneously. As the mechanosensing amplitude increases, the division number increases. That is the case because ΔV is bounded by 0, meaning cells can only grow or arrest their cell cycle, making the ΔV contribution due to mechanosensing biased to be positive. **D)** Average cell area versus time for different mechanosensing amplitudes.
(TIF)

S2 Fig T1 transitions during the growth process and cell trajectories between two successive avalanches. A) T1 transitions during the growth process of the epithelial sheet. There is no significant correlation between avalanche onset and occurrence of T1 transitions. The movement of cells recorded after an avalanche at *T* = 44.4*h* for trajectory displacement B) and trajectory angles in C). Cell movement does not exhibit the collective behavior seen during an avalanche. There are disparate regions of the tissue where cells exhibit significant displacement and the boundary does not significantly expand. The formation of these regions and the inhomogeneity of the system are indicative of inhomogeneous accumulation of stress that leads to avalanches.
(TIF)

S3 Fig Standard deviation of the radial average cell area right before an avalanche versus the tissue area change after the onset of the given avalanche. The y-axis provides a measure of cell packing inhomogeneity. The correlation was found to be weak but non-negligible, with a spearman’s rank correlation coefficient of *ρ* = 0.16 and a *p*<0.01.
(TIF)

S4 Fig Fitting considerations. **A)** Log-log plot of the probability density versus tissue jump (or avalanche) magnitude. The coefficient was obtained to be *τ* = 1.25 by a linear fit. The power law has an exponential cutoff due to finite system size. **B)** Semi-log plot for probability density versus tissue jump magnitude. The linear fit is good and suggests that the distribution may exhibit an exponential signature. However, as shown in S5 Fig, the exponential fit is poor when extracting the exponent after varying model parameters.
(TIF)

S5 Fig Exponent dependence on parameter variation. **A)** Log-log plot of the probability density versus tissue jump magnitude when T1 transitions are disabled. The slope coefficient was obtained to be invariant with *τ* = 1.27. **B)** Log-log plot of the probability density versus tissue jump magnitude when mechanosensing amplitude, μ, is one quarter of the original value T1 transitions are disabled. The slope coefficient was obtained to be invariant with *τ* = 1.27. **C&D)** Semi-log plots for probability density versus tissue jump magnitude for A and B respectively. The linear fit is poor and suggests that the distributions do not exhibit an exponential signature.
(TIF)

S6 Fig Effect of apical constriction on MF speed and cell packing. **A)** The MF speed decreases as apical constriction increases. **B)** The cellular mosaic becomes increasingly packed with increasing apical constriction. This speed dependence on apical constriction is a consequence of the signaling differential equations, which are discretized on the cellular lattice, and are independent of the physical distance between cells. The blue dots involve data taken from simulations were the MF reached the anterior portion and the growth process was concluded. The red dots indicate data from simulations that were not completed since the tissue reaches a very large number of cells, making them very computationally costly.
(TIF)

S7 Fig Boundary tension analysis. **A)** Circularity index, defined as *c*_0_ = 4*πAP*^−2^, where A is the tissue area and P the perimeter. The tissue became increasingly circular as the boundary tension was increased. The effect of increasing boundary tension is more prominent for low values and saturates ~1 for larger values. Boundary tension value used throughout this paper was set to 0.7. **B)** Cell number versus time. The changes in boundary tension do not significantly affect the growth rate of the tissue.
(TIF)

S8 Fig Uniform growth case with overdamped dynamics: Tissue area and cell area using a viscous solver, under different viscosity factors. **A)** Tissue area vs time. **B)** Cell area vs time. No matter which viscosity factor we choose, the tissue grows smoothly, without avalanches. The cell area decreases at later stages for all cases, as cells cannot displace fast enough to accommodate the exponential growth of the tissue. As the viscosity becomes smaller (inverse viscosity becomes larger), the tissue area and cell area tend to be larger. Also, the cell areas are smoother when the viscosity is smaller. Note that the viscosity units are arbitrary, however the vast range allows us to probe the range of low and high viscosity dynamics. For each structural update, the cellular vertices were displaced in a resolution of 100 steps with a magnitude and direction set by the force experienced at each step.
(TIF)

S9 Fig Uniform growth case: Avalanches in hexagonal and soft cellular networks. By Incrementally decreasing the bulk tension in the system, in accordance with (32), the tissue structure goes through a phase transition, from a hexagonal network to a soft network phase. We find the transition point from hexagonal network to soft network is -0.33. If the tension is larger than this value, the tissue lies in the hexagonal network phase, otherwise it is in the soft network phase, with larger cell area and sharp shaped cells. **A)** Tissue area vs time. **B)** Cell area vs time. **C)** Zoomed in plot of tissue area vs time (Soft network) **D)** Zoomed in plot of cell area vs time (Soft network). We find that the avalanches are absent when transitioning to the soft network, as expected because cells become easily deformable and prevent proliferative stress buildup in the soft network phase.
(TIF)
