# Supplementary figures and images for: Avalanches during epithelial tissue growth; Uniform Growth and a drosophila eye disc model
Source: PLoS Comput Biol. 2022 Mar 18;18(3):e1009952. doi: 10.1371/journal.pcbi.1009952 (PMC8932575; doi:10.1371/journal.pcbi.1009952)

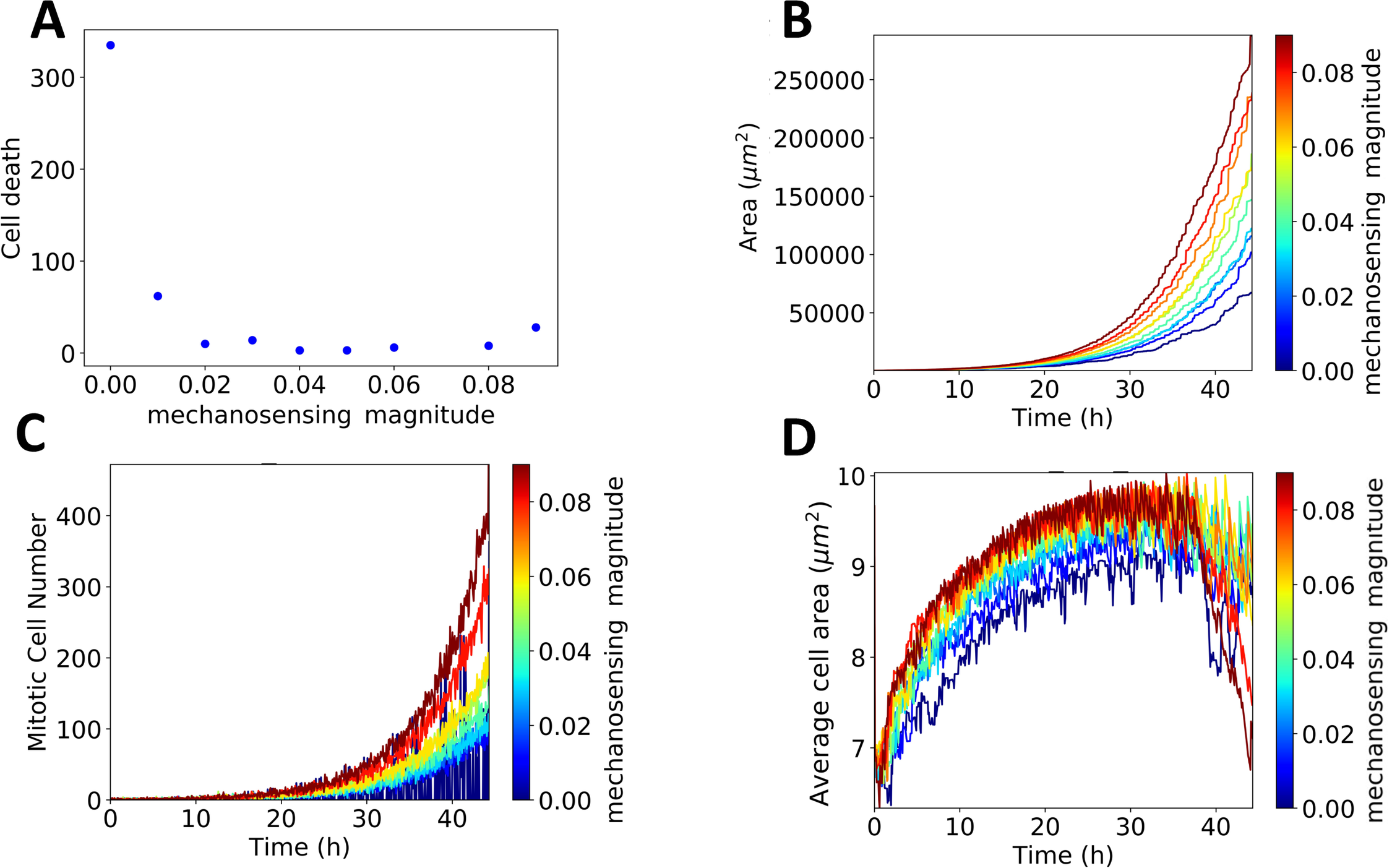

Supplement: S1 Fig — A: Cell death during growth for different mechanosensing amplitudes. When mechanosensing is toggled off, at zero mechanosensing magnitude (μ), there is significant cell death due to proliferation stresses. When non-zero values of mechanosensing are used, cell death becomes negligible. B: Area versus time for different mechanosensing amplitudes. The mechanosensing amplitude significantly affects the growth process. Since this is exponential growth, small variations intensify exponentially. Close to μ = 0.04, which is the parameter used and is taken from [29], the area does not deviate when changing mechanosensing. Growth discontinuities or avalanches are present for all mechanosensing parameters. C) Division number versus time for variable mechanosensing magnitudes. When mechanosensing is turned off, the cells divide periodically since all daughter cells grow instantaneously. As the mechanosensing amplitude increases, the division number increases. That is the case because ΔV is bounded by 0, meaning cells can only grow or arrest their cell cycle, making the ΔV contribution due to mechanosensing biased to be positive. D) Average cell area versus time for different mechanosensing amplitudes. (TIF) [file pcbi.1009952.s007.tif]

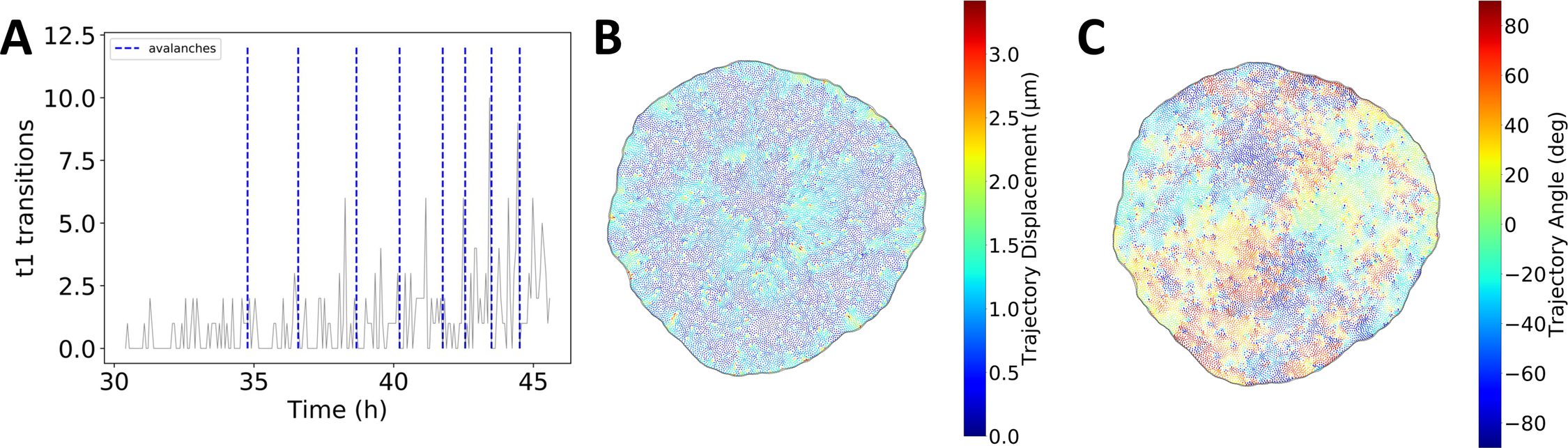

Supplement: S2 Fig — A) T1 transitions during the growth process of the epithelial sheet. There is no significant correlation between avalanche onset and occurrence of T1 transitions. The movement of cells recorded after an avalanche at T = 44.4h for trajectory displacement B) and trajectory angles in C). Cell movement does not exhibit the collective behavior seen during an avalanche. There are disparate regions of the tissue where cells exhibit significant displacement and the boundary does not significantly expand. The formation of these regions and the inhomogeneity of the system are indicative of inhomogeneous accumulation of stress that leads to avalanches. (TIF) [file pcbi.1009952.s008.tif]

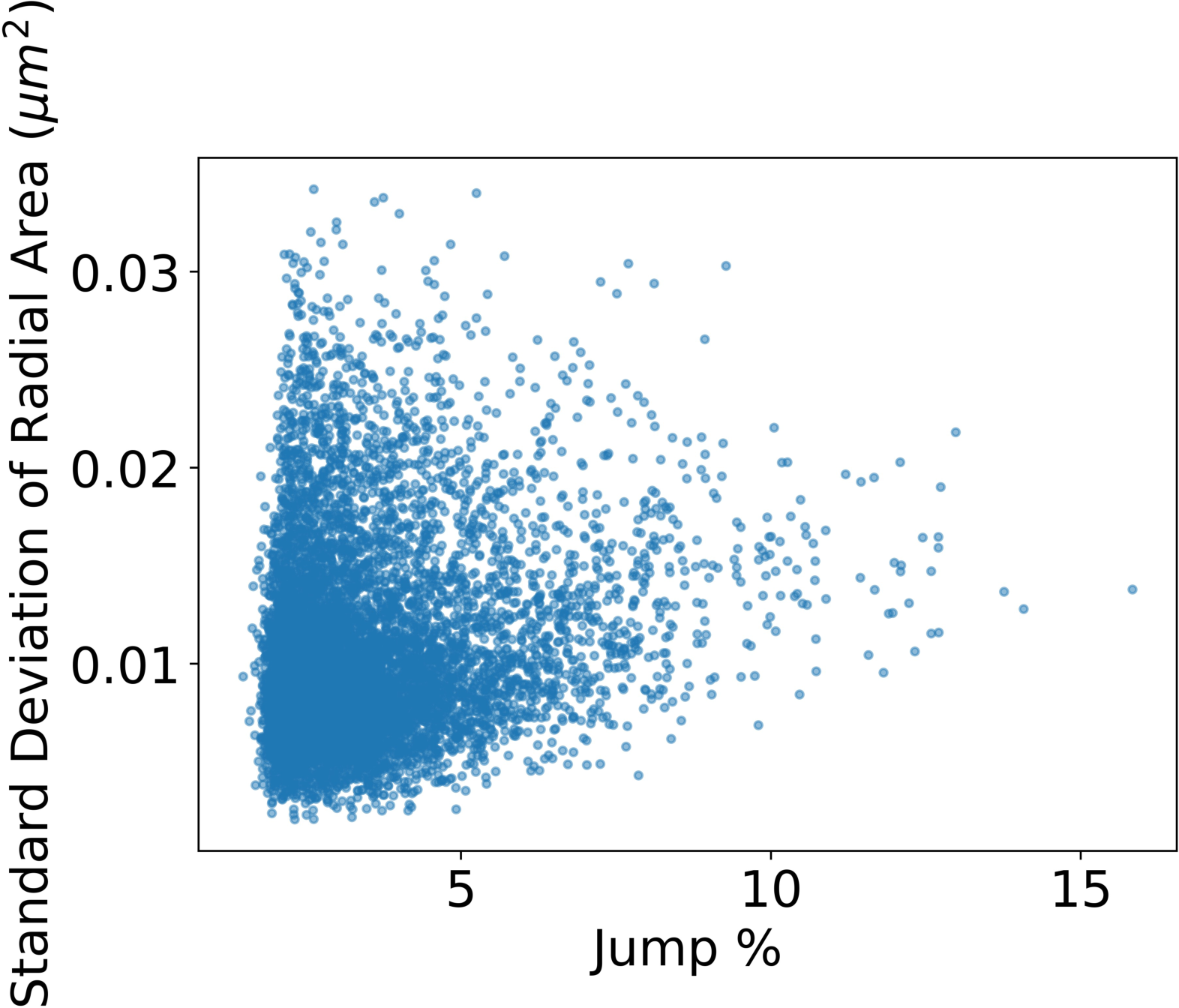

Supplement: S3 Fig — The y-axis provides a measure of cell packing inhomogeneity. The correlation was found to be weak but non-negligible, with a spearman’s rank correlation coefficient of ρ = 0.16 and a p<0.01. (TIF) [file pcbi.1009952.s009.tif]

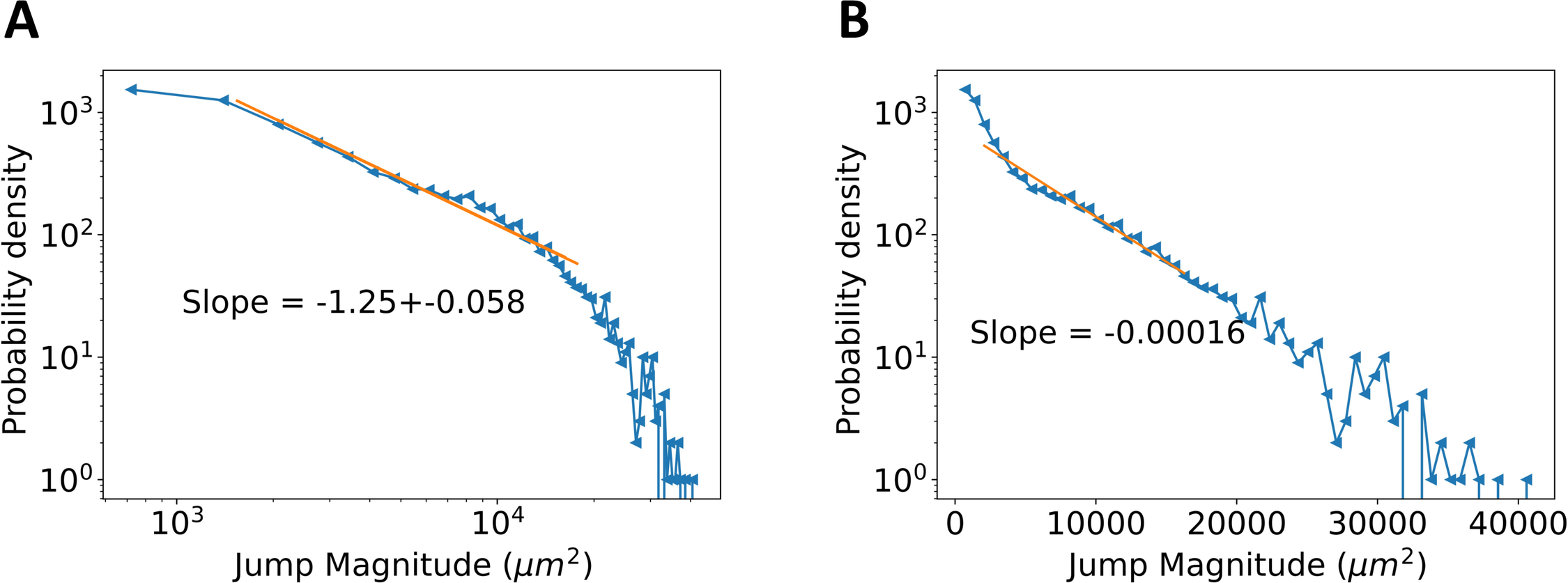

Supplement: S4 Fig — A) Log-log plot of the probability density versus tissue jump (or avalanche) magnitude. The coefficient was obtained to be τ = 1.25 by a linear fit. The power law has an exponential cutoff due to finite system size. B) Semi-log plot for probability density versus tissue jump magnitude. The linear fit is good and suggests that the distribution may exhibit an exponential signature. However, as shown in S5 Fig, the exponential fit is poor when extracting the exponent after varying model parameters. (TIF) [file pcbi.1009952.s010.tif]

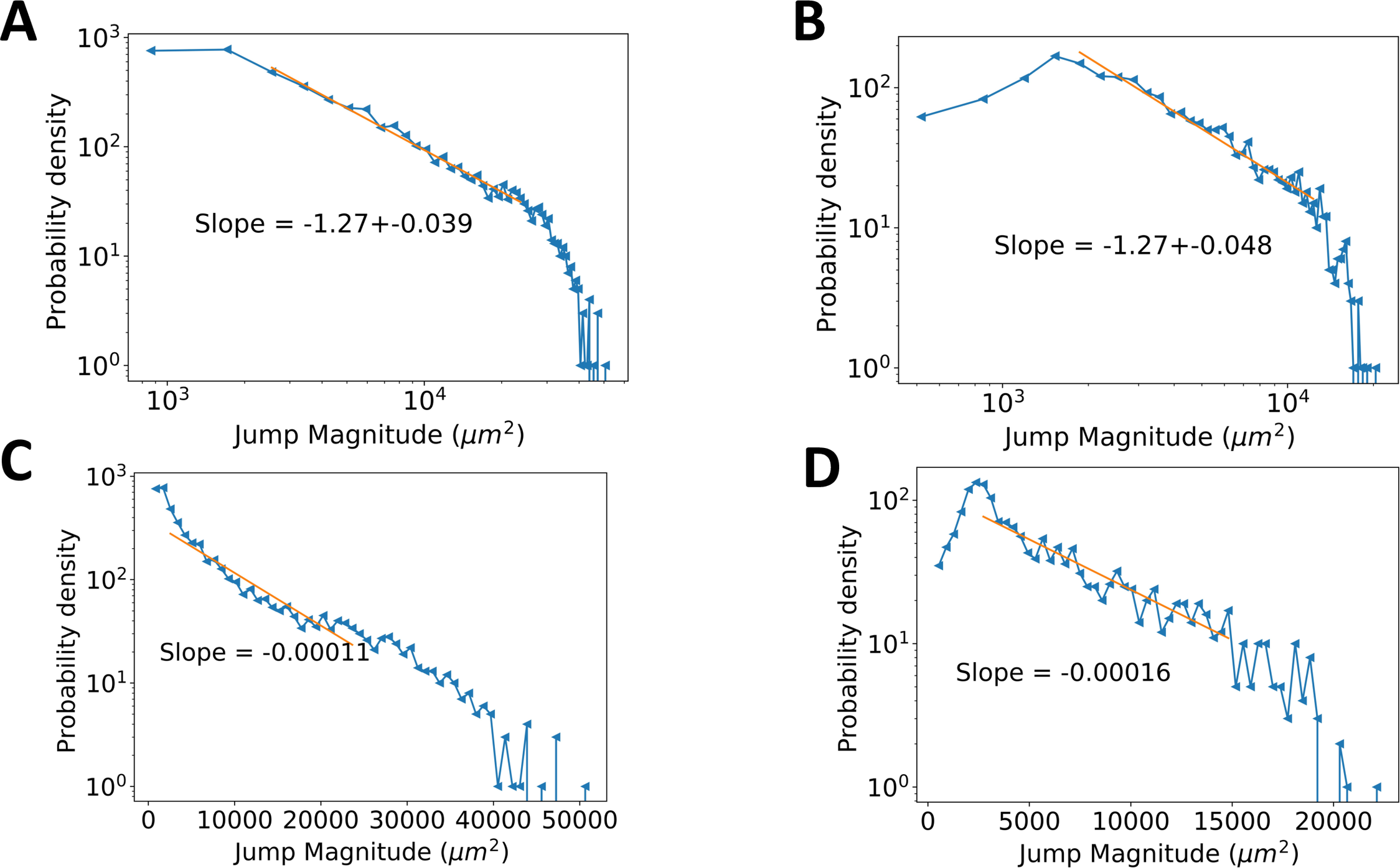

Supplement: S5 Fig — A) Log-log plot of the probability density versus tissue jump magnitude when T1 transitions are disabled. The slope coefficient was obtained to be invariant with τ = 1.27. B) Log-log plot of the probability density versus tissue jump magnitude when mechanosensing amplitude, μ, is one quarter of the original value T1 transitions are disabled. The slope coefficient was obtained to be invariant with τ = 1.27. C&D) Semi-log plots for probability density versus tissue jump magnitude for A and B respectively. The linear fit is poor and suggests that the distributions do not exhibit an exponential signature. (TIF) [file pcbi.1009952.s011.tif]

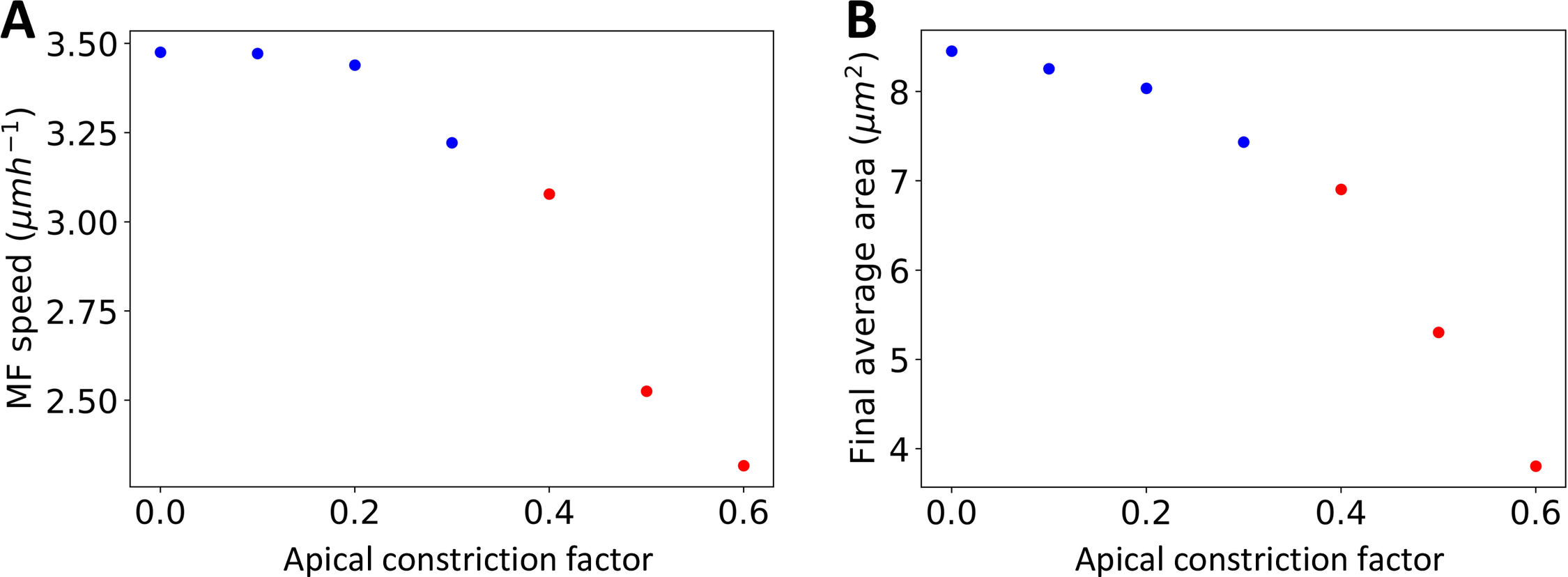

Supplement: S6 Fig — A) The MF speed decreases as apical constriction increases. B) The cellular mosaic becomes increasingly packed with increasing apical constriction. This speed dependence on apical constriction is a consequence of the signaling differential equations, which are discretized on the cellular lattice, and are independent of the physical distance between cells. The blue dots involve data taken from simulations were the MF reached the anterior portion and the growth process was concluded. The red dots indicate data from simulations that were not completed since the tissue reaches a very large number of cells, making them very computationally costly. (TIF) [file pcbi.1009952.s012.tif]

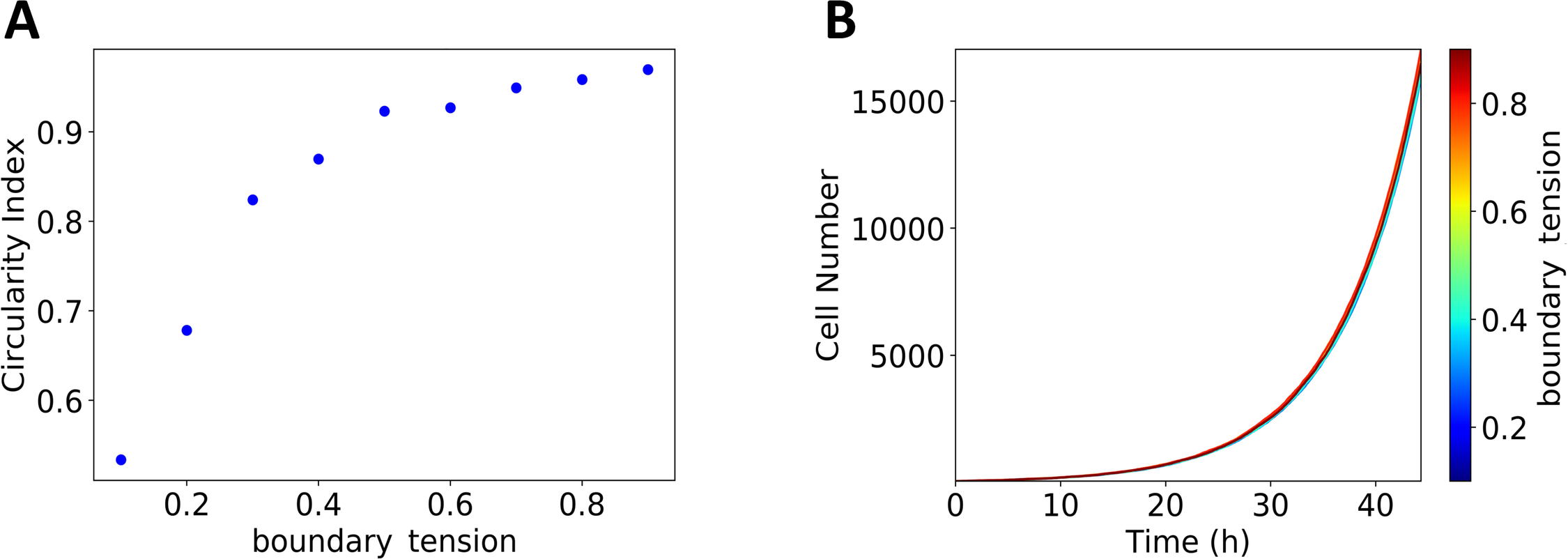

Supplement: S7 Fig — A) Circularity index, defined as c0 = 4πAP−2, where A is the tissue area and P the perimeter. The tissue became increasingly circular as the boundary tension was increased. The effect of increasing boundary tension is more prominent for low values and saturates ~1 for larger values. Boundary tension value used throughout this paper was set to 0.7. B) Cell number versus time. The changes in boundary tension do not significantly affect the growth rate of the tissue. (TIF) [file pcbi.1009952.s013.tif]

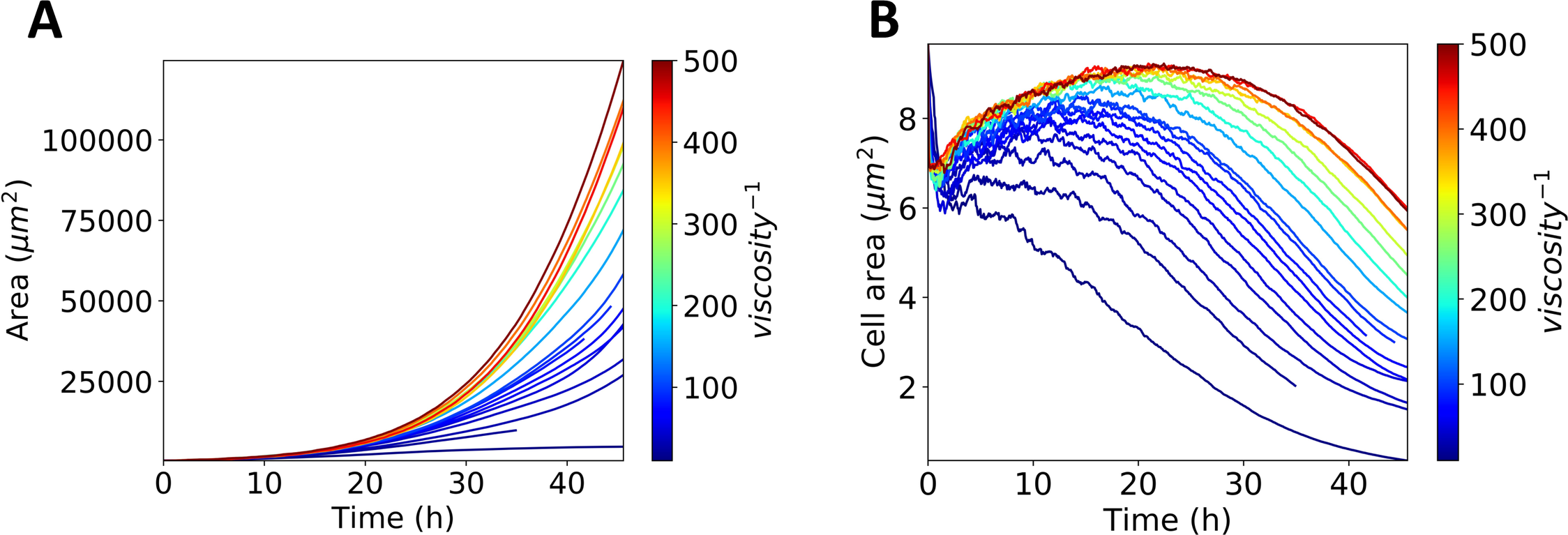

Supplement: S8 Fig — A) Tissue area vs time. B) Cell area vs time. No matter which viscosity factor we choose, the tissue grows smoothly, without avalanches. The cell area decreases at later stages for all cases, as cells cannot displace fast enough to accommodate the exponential growth of the tissue. As the viscosity becomes smaller (inverse viscosity becomes larger), the tissue area and cell area tend to be larger. Also, the cell areas are smoother when the viscosity is smaller. Note that the viscosity units are arbitrary, however the vast range allows us to probe the range of low and high viscosity dynamics. For each structural update, the cellular vertices were displaced in a resolution of 100 steps with a magnitude and direction set by the force experienced at each step. (TIF) [file pcbi.1009952.s014.tif]

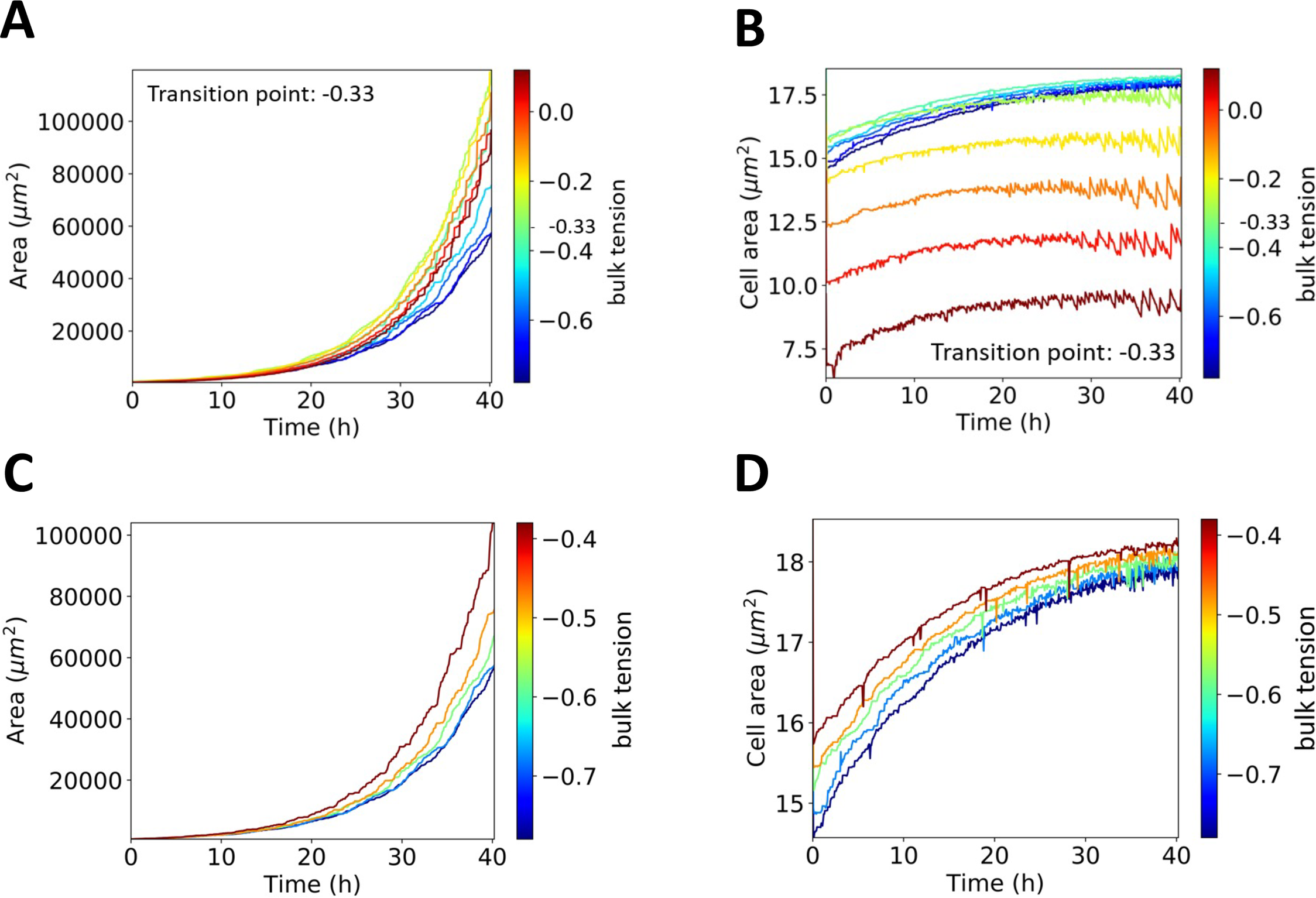

Supplement: S9 Fig — By Incrementally decreasing the bulk tension in the system, in accordance with (32), the tissue structure goes through a phase transition, from a hexagonal network to a soft network phase. We find the transition point from hexagonal network to soft network is -0.33. If the tension is larger than this value, the tissue lies in the hexagonal network phase, otherwise it is in the soft network phase, with larger cell area and sharp shaped cells. A) Tissue area vs time. B) Cell area vs time. C) Zoomed in plot of tissue area vs time (Soft network) D) Zoomed in plot of cell area vs time (Soft network). We find that the avalanches are absent when transitioning to the soft network, as expected because cells become easily deformable and prevent proliferative stress buildup in the soft network phase. (TIF) [file pcbi.1009952.s015.tif]
